# Providing Groceries and Transportation to Poverty-Exposed Pediatric Oncology Families: The PediCARE Pilot Randomized Clinical Trial

**DOI:** 10.1001/jamanetworkopen.2024.12890

**Published:** 2024-05-31

**Authors:** Haley Newman, Emily Jones, Yimei Li, Puja J. Umaretiya, Julie A. Wolfson, Joanne Wolfe, Kira Bona

**Affiliations:** 1 Department of Pediatrics, Division of Oncology, Children’s Hospital of Philadelphia, Philadelphia, Pennsylvania; 2 Department of Pediatrics, Division of Pediatric Hematology/Oncology, Boston Children’s Hospital, Boston, Massachusetts; 3 Department of Biostatistics, Epidemiology, and Informatics, University of Pennsylvania Perelman School of Medicine, Philadelphia; 4 Division of Pediatric Hematology and Oncology, University of Texas Southwestern Medical Center, Dallas; 5 Division of Pediatric Hematology-Oncology, University of Alabama at Birmingham, Birmingham; 6 Institute for Cancer Outcomes and Survivorship, University of Alabama at Birmingham, Birmingham; 7 Department of Pediatrics, Massachusetts General Hospital, Boston; 8 Department of Pediatrics, Brigham and Women’s Hospital, Boston, Massachusetts; 9 Department of Pediatric Oncology and Division of Population Sciences, Dana-Farber Cancer Institute, Boston, Massachusetts

## Abstract

This randomized clinical trial evaluates the Pediatric Cancer Resource Equity (PediCARE) intervention, which provided groceries and transportation, vs usual care, for poverty-exposed pediatric oncology families.

## Introduction

Poverty is independently associated with childhood cancer relapse and death.^1,2^ To date, no evidence-based interventions targeting poverty as a risk factor for poor outcome have been evaluated in pediatric oncology. We evaluated central administration of the Pediatric Cancer Resource Equity (PediCARE) intervention,^3^ which provided groceries and transportation to poverty-exposed pediatric oncology families. The trial aimed to assess feasibility data necessary to inform randomized efficacy trials, including (1) caregiver willingness to be randomized to a poverty-targeted intervention and (2) attrition among participants randomized to standard of care.

## Methods

This pilot randomized clinical trial of usual supportive care (control) vs usual supportive care plus PediCARE (intervention) included participants at Dana-Farber Cancer Institute (DFCI; Boston, Massachusetts) and University of Alabama at Birmingham (UAB) enrolled between May 2019 and August 2021. Children aged 0 to 17 years within 2 months of a de novo cancer diagnosis with planned receipt of at least 4 cycles of chemotherapy were eligible if their parent and/or guardian self-reported household material hardship (HMH), defined as at least 1 domain of food, housing, utility, or transportation insecurity. Parent and/or guardians provided consent for study enrollment on behalf of their children. This study was approved by the DFCI institutional review board (IRB), which served as the central IRB for both enrolling institutions, and was registered on ClinicalTrials.gov (NCT03638453). This report follows the CONSORT reporting guideline for pilot or feasibility randomized trials.

Participants were randomized 1:1, stratified by site, to control vs intervention for 6 months. Intervention participants received PediCARE, a centrally administered standardized allocations of groceries via the Instacart online grocery-delivery platform, and transportation via patient-scheduled ride-share or gas cards, in addition to usual supportive care. Participation included baseline and 6-month parent and/or guardian surveys. Trial protocol, statistical design, and surveys are provided in Supplement 1. Additional methods are shown in the eAppendix in Supplement 2. The primary study end point was feasibility, defined as more than 75% consent to randomization or participation, and less than 20% attrition (study withdrawal or incomplete 6-month time point survey) per group.

## Results

Among 41 families (28 at DFCI and 13 at UAB) (Table), 100% consented to enrollment and randomization; 1 patient at UAB was subsequently deemed ineligible (Figure). There was 0% attrition in either group, and 100% baseline and 6-month survey completion. All intervention participants successfully received grocery and/or transportation resources.

**Table.** Patient, Caregiver, and Household Characteristics by Treatment Group

Characteristics	Participants, No. (%)		
	Overall (N = 40)	PediCARE intervention (n = 20)	Usual care (n = 20)
Caregivers			
Relationship to patient			
Mother	35 (87.5)	15 (75.0)	20 (100.0)
Father	4 (10.0)	4 (20.0)	0
Legal guardian	1 (2.5)	1 (5.0)	0
Primary language			
English	25 (62.5)	12 (60.0)	13 (65.0)
Spanish	7 (17.5)	5 (25.0)	2 (10.0)
Other^a^	8 (20.0)	3 (15.0)	5 (25.0)
Marital status			
Single, separated, or divorced	20 (50.0)	8 (40.0)	12 (60.0)
Married or living with partner	20 (50.0)	12 (60.0)	8 (40.0)
Highest education			
High school or less	20 (50.0)	9 (45.0)	11 (55.0)
Trade or vocational school or some college	11 (28.0)	7 (35.0)	4 (20.0)
College graduate	9 (22.5)	4 (20.0)	5 (25.0)
Patients			
Age, mean (SD), y	8.6 (5.9)	8.9 (6.3)	8.3 (5.8)
Ethnicity^b^			
Hispanic	12 (30.0)	7 (35.0)	5 (25.0)
Not Hispanic	28 (70.0)	13 (65.0)	15 (75.0)
Race^b^			
Asian	2 (5.0)	1 (5.0)	1 (5.0)
Black	17 (42.5)	8 (40.0)	9 (45.0)
White	20 (50.0)	10 (50.0)	10 (50.0)
Missing	1 (2.5)	1 (5.0)	0
Households			
Annual household income, median (range), $	27 300 (700-80 000)	30 000 (1500-80 000)	26 160 (700-80 000)
Low income^c^			
Yes	26 (65.0)	12 (60.0)	14 (70.0)
No	4 (10.0)	1 (5.0)	3 (15.0)
Missing	10 (25.0)	7 (35.0)	3 (15.0)
No. of domains of HMH			
1	10 (25.0)	6 (30.0)	4 (20.0)
2	13 (32.5)	5 (25.0)	8 (40.0)
3	11 (27.5)	5 (25.0)	6 (30.0)
4	6 (15.0)	4 (20.0)	2 (10.0)
HMH domains			
Food	30 (75.0)	17 (85.0)	13 (65.0)
Housing	28 (70.0)	14 (70.0)	14 (70.0)
Transportation	14 (35.0)	7 (35.0)	7 (35.0)
Utilities	21 (53.0)	9 (45.0)	12 (60.0)
Participation in public support programs			
Medicaid	33 (82.5)	19 (95.0)	14 (70.0)
Supplemental Nutrition Assistance Program	22 (55.0)	12 (60.0)	10 (50.0)
Free school lunch	20 (50.0)	11 (55.0)	9 (45.0)
Food pantry	8 (20.0)	3 (15.0)	5 (25.0)
Energy assistance	8 (20.0)	4 (20.0)	4 (20.0)
Disability benefits	6 (15.0)	3 (15.0)	3 (15.0)
Public housing	8 (20.0)	5 (25.0)	3 (15.0)

Abbreviation: HMH, household material hardship.

^a^ Includes Arabic, Portuguese, Haitian-Creole, Cambodian, French, Quiché, and Vietnamese.

^b^ Children’s race and ethnicity were self-reported by parents and guardians and were included to demonstrate the distribution of minoritized patients in this cohort.

^c^ Low income is defined as less than 200% of the federal poverty level for the year 2020.

**Figure.**
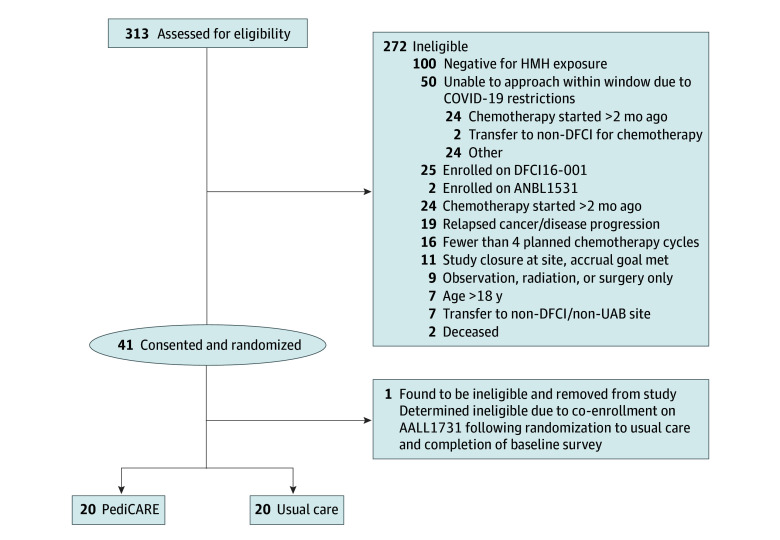
CONSORT Diagram ANBL indicates Children’s Oncology Group study ANBL1531; DFCI, Dana-Farber Cancer Institute; HMH, household material hardship; UAB, University Alabama Birmingham.

A greater proportion of intervention participants experienced stabilized (no change) or decreased numbers of HMH domains at 6 months vs control (17 participants [85%] vs 14 participants [70%]). More intervention than control participants reported no HMH at 6 months (5 participants [25%] vs 2 participants [10%]).

## Discussion

In this randomized clinical trial, 100% of participants consented to randomization with 0% attrition in either group. These data demonstrate caregiver recognition of the need for evidence-based interventions to address poverty during childhood cancer treatment and a willingness to participate in randomized research to evaluate such interventions.

We successfully enrolled a population of families facing multiple social risks, supporting the feasibility of health equity intervention evaluation among historically marginalized subspecialty populations. Equally important, we demonstrate the feasibility of a centrally delivered intervention a priori designed for scalability. Owing to the rarity of childhood cancer, most US children receive treatment via multicenter clinical drugs trials that accrue at more than 200 centers to achieve adequate samples sizes for statistical power.^4^ Evaluation of disease-specific health equity intervention efficacy in pediatric oncology can only be accomplished in this multicenter trial setting, requiring interventions designed to scale with minimal site burden. This pilot study was unable to detect statistically significant differences in HMH between arms. Although it was conducted in hospitals in diverse geographic settings, feasibility findings may not be generalizable to all sites of pediatric oncology care.

One in 3 children with cancer is exposed to poverty,^5,6^ and these children are more likely to relapse and die. Ensuring equity in pediatric oncology requires evaluation of novel interventions targeting this independent risk factor. These data lay the groundwork for systematic incorporation of poverty-targeted interventions in future pediatric oncology trials.

## Notes

Supplement 1.
Trial Protocol and Statistical Analysis Plan

Supplement 2. **eAppendix.** Supplemental Methods

Supplement 3.
Data Sharing Statement

## References

[1] Bona K, Li Y, Winestone LE, . Poverty and targeted immunotherapy: survival in Children’s Oncology Group clinical trials for high-risk neuroblastoma. J Natl Cancer Inst. 2021;113(3):282-291. doi:10.1093/jnci/djaa107 33227816 PMC7936051

[2] Gupta S, Dai Y, Chen Z, . Racial and ethnic disparities in childhood and young adult acute lymphocytic leukaemia: secondary analyses of eight Children’s Oncology Group cohort trials. Lancet Haematol. 2023;10(2):e129-e141. doi:10.1016/S2352-3026(22)00371-4 36725118 PMC9951049

[3] Umaretiya PJ, Revette A, Seo A, . PediCARE: development of a poverty-targeted intervention for pediatric cancer. Pediatr Blood Cancer. 2021;68(10):e29195. doi:10.1002/pbc.29195 34190405 PMC8384686

[4] Hawkins DS, Gore L. Children’s Oncology Group’s 2023 blueprint for research. Pediatr Blood Cancer. 2023;70(suppl 6)(suppl 6):e30569. doi:10.1002/pbc.30569 37433635 PMC10529891

[5] Umaretiya PJ, Koch VB, Flamand Y, . Disparities in parental distress in a multicenter clinical trial for pediatric acute lymphoblastic leukemia. J Natl Cancer Inst. 2023;115(10):1179-1187. doi:10.1093/jnci/djad099 37261858 PMC10560600

[6] Karvonen KA, Umaretiya PJ, Koch VB, . Inequitable poverty exposures: a subspecialty opportunity to address disparities. Hosp Pediatr. 2024;14(2):e104-e106. doi:10.1542/hpeds.2023-007482 38239110 PMC10823182

