# Supplementary material for: Providing Groceries and Transportation to Poverty-Exposed Pediatric Oncology Families: The PediCARE Pilot Randomized Clinical Trial
Source: JAMA Netw Open. 2024 May 31;7(5):e2412890. doi: 10.1001/jamanetworkopen.2024.12890 (PMC11143457; doi:10.1001/jamanetworkopen.2024.12890)
Supplement: Supplement 1. — Trial Protocol and Statistical Analysis Plan [file jamanetwopen-e2412890-s001.pdf]

PROTOCOL TITLE:

Pilot Feasibility of the Pediatric Cancer Resource Equity (PediCARE) Intervention

PRINCIPAL INVESTIGATOR:

Kira Bona, MD MPH
Department of Pediatric Oncology
617-632-4688
Kira_Bona@dfci.harvard.edu

VERSION NUMBER:

1.6

DATE:

May 24, 2021

Abbreviations

AYA	Adolescents & young adults
CRA	Clinical research assistant
DSMP	Data and safety monitoring plan
HMH	Household material hardship
HMHS	Household material hardship survey
SDMC	Survey & data management core
UAB	University of Alabama Birmingham
UC	Usual care

Table of Contents

1.0 Background 4
2.0 Objectives 4
2.1 Overall Study Objectives: 4
2.2 Specific Aims: 5
3.0 Inclusion and Exclusion Criteria 5
4.0 Planned Enrollment 6
5.0 Study-Wide Recruitment Methods 6
5.1 Recruitment: 6
5.2 Consent Process: 7
5.3 Registration Process: 7
5.4 Strategies for retention: 8
6.0 Multi-Site Research 8
7.0 Study Timelines 9
8.0 Study Endpoints 9
9.0 Study Procedures 10
9.1 Overall Study Design 10
9.2 Procedures for Assignment to Study Group: 12
9.3 Intervention Procedures: 12
10.0 Data Management and Confidentiality 14
11.0 Statistical Considerations 15
12.0 Data Monitoring Provisions to Ensure the Safety of Subjects 15
13.0 Risks to Subjects 15
14.0 Potential Benefits to Subjects 17
15.0 Sharing of Results with Subjects 17
16.0 Study Evaluations Table 18
17.0 References 19
18.0 Appendices 22
Appendix A. Grocery Allocation Household Size Table 23
Appendix B. Parent Surveys (Baseline and Post-Intervention) 24
Appendix C. Instacart Information Sheet 65
Appendix D. Data Safety Monitoring Plan 68
Appendix E. Ride Health Information Sheet 78

1.0 Background

One in five children diagnosed with cancer in the U.S. lives in poverty;[1] and poor children with cancer experience higher risks of relapse and decreased overall survival.[2,3] We previously found that in the context of a clinical trial, children with acute lymphoblastic leukemia from high-poverty neighborhoods experienced lower 5-year overall survival (85% vs 92%; p=0.02) and higher rates of early relapse (92% vs 48%, p=0.008) which is more challenging to cure.[4] Despite these disparities, poverty as a contributor to disease outcome has never been targeted in a systematic fashion in childhood cancer. We hypothesize that interventions targeting modifiable domains of poverty during cancer therapy will reduce disparities in childhood cancer outcomes.

One barrier to the development of poverty-targeted interventions for the clinical setting is the perception of poverty as a non-modifiable variable. Household material hardship (HMH), a concrete measure of poverty defined as unmet basic needs including food, heat, housing or transportation, represents an ideal focus for poverty-targeted intervention. HMH is both associated with inferior child health outcomes and remediable with intervention.[5-9] Notably, prior work by our group has identified HMH in 20% of pediatric cancer families at diagnosis and 30% after the initial six months of chemotherapy.[10] Multiple trials in primary care have demonstrated that systematic screening for HMH and referral to existing governmental and community resources (e.g. food stamps or food bank) both reduce HMH and improve health outcomes—including vaccination rates in children, and hypertension and hypercholesterolemia in adults.[9,11,12] While these data provide proof of principle that poverty-targeted interventions impact health outcomes, we hypothesize that the time-sensitive and life-threatening nature of a childhood cancer diagnosis requires more immediately impactful interventions that build upon systematic screening and referral.

In preliminary qualitative work, we identified food and transportation insecurity as domains of HMH most challenging for pediatric families during cancer therapy. We subsequently developed the Pediatric Cancer Resource Equity (PediCARE) intervention, an HMH-intervention with novel, scalable components targeting food and transportation insecurity with the direct provision of these resources. We refined the PediCARE intervention by pretesting it in a small cohort of newly diagnosed pediatric cancer families with pre-existing HMH (DFCI Protocol 18-275) and gathering qualitative and quantitative parent-feedback on intervention design and conduct. We will now evaluate the feasibility of the refined PediCARE intervention in a pilot randomized trial at two-centers. Data from this feasibility pilot will lay the groundwork for a subsequent multi-center study of PediCARE with a goal of reducing HMH and improving child outcomes.

2.0 Objectives

2.1 Overall Study Objectives:

The overarching goal of this study is to pilot a randomized controlled trial (RCT) of the Pediatric Cancer Resource Equity (PediCARE) intervention to determine feasibility of intervention administration in a childhood cancer population. Secondary goals include generation of effect size estimates for a primary endpoint of reduction in household material hardship (HMH) and a secondary endpoint of Emergency Department and Intensive Care Unit utilization to support

planning of a subsequent definitive multi-center RCT. PediCARE is a poverty-targeted intervention utilizing direct resource provision of groceries and transportation.

2.2 Specific Aims:

Pilot RCT to determine feasibility

To conduct a pilot RCT of the PediCARE intervention in 40 newly diagnosed pediatric cancer families with HMH at DFCI and UAB. Families in the intervention arm will receive PediCARE for 6-months. The control group will receive usual care. Data will be collected by parent survey at baseline and 6-months. The primary outcome will be defined as feasibility. Secondary outcomes will include improvement in HMH scores (range 0-4) at 6-months compared to baseline and Emergency Department (ED) and Intensive Care Unit (ICU) admissions. Analyses will focus on effect size estimates to plan a subsequent RCT.
H2a. Feasibility will be demonstrated by recruitment rate of 75% and follow-up survey data collection of 80% per arm.
H2b: There will be a trend towards a reduction in HMH scores (primary endpoint) and lower proportion of patients experiencing an ED visit or ICU admission over 6-months in the PediCARE versus usual care group.

3.0 Inclusion and Exclusion Criteria

Children diagnosed with cancer at DFCI and UAB will serve as the study cohort for PediCARE with parents/guardians as survey informants and intervention recipients.

3.1 Inclusion criteria:

1) Child initiated chemotherapy in the last 2 months for newly diagnosed cancer;
2) Planned receipt of at least 4 cycles of chemotherapy at DFCI or UAB;
3) Parent/guardian screened positive for HMH*;
4) Child is <18 years at time of enrollment

* In accordance with previous research[15-17,21,42] families will be operationalized as having HMH for eligibility purposes if they report at least one of the below four concrete needs: (1) Food insecurity.[47.39,48] (2) Housing Insecurity.[16,17](3) Energy Insecurity.[16,17] (4) Transportation Insecurity. Families are considered to have insecurity in a domain if they screen positive to at least one item. HMH screening is performed as standard of care by site-specific providers or will be conducted by a member of the research study team.

3.2 Exclusion criteria:

1) Child with diagnosis of relapsed cancer;
2) Child planned to receive fewer than 4 cycles of chemotherapy
3) Child planned to receive observation, radiation or surgical resection only;
4) Planned transfer of child to a non-DFCI or UAB facility for chemotherapy treatment;
5) Foreign national family receiving cancer care as an Embassy-pay patient;
6) Child is enrolled on DFCI 16-001 (due to ongoing embedded descriptive HMH study) or NA if enrolling at UAB

7) Child is enrolled on ANBL1531 or AALL1731 (due to ongoing embedded descriptive HMH studies

3.3 Inclusion of Women and Minorities:
This study focuses on children with a new diagnosis of cancer with their parents as survey informants and recipients of the PediCARE intervention. This study will enroll racial and ethnic minorities in a manner that is reflective of the proportion of racial and ethnic minorities diagnosed with childhood cancer at each participating site (DFCI and UAB). This study does not focus on any race, ethnicity, or gender. No potential research subjects will be excluded from enrollment based on race, ethnic origin, or gender; nor will any race, ethnicity or gender be preferentially enrolled. All children and their families receiving chemotherapy at DFCI or UAB who meet the primary inclusion criteria of screening positive for household material hardship (HMH) at the time of diagnosis will be considered equally eligible for participation. It is notable that this is a poverty-targeted intervention, and thus based on the epidemiology of poverty in the United States racial and ethnic minorities may well be overrepresented in a cohort selected for pre-existing poverty at the time of diagnosis.

3.4 Inclusion of Children:
This study is a poverty-targeted intervention pilot for pediatric cancer families. Consequently, children diagnosed with cancer at DFCI and UAB will serve as the study cohort for PediCARE with parents/guardians as survey informants and intervention recipients. The rationale for the inclusion of children in this intervention pilot is that our overall research agenda is to improve childhood cancer outcomes utilizing poverty-targeted interventions during therapy.
Child involvement in this study will be limited to descriptive medical record abstraction of disease, treatment, and care utilization data. Children will not experience direct study contact or intervention, as such it will represent minimal risk.

4.0 Planned Enrollment

This study aims to enroll a total of 40 participants as follows:

Pilot RCT DFCI and UAB: The target sample size is 40 participants. At DFCI, a total of 28 participants will be enrolled and randomized (thus 14 participants will receive PediCARE and 14 participants will receive UC). At UAB a total of 12 participants will be enrolled and randomized (thus 6 participants will receive PediCARE and 6 participants will receive UC).

Families will be recruited consecutively at each site over a 6-month time period. Patients will be randomized 1:1 to PediCARE versus Usual Care (UC) and randomization will be stratified by site.

5.0 Study-Wide Recruitment Methods

5.1 Recruitment:
New patients at each site (DFCI and UAB) are continuously identified by a local study teams (including clinical providers and trained clinical research assistants (CRAs)) and screened for eligibility to all open research protocols. Study teams will email the primary oncology team for all

PediCARE-eligible patients requesting permission to approach in-line with the standard research practice in Pediatric Oncology. We will reduce gate-keeping effects by educating providers about high family satisfaction with participation in prior HMH studies by our group. The providers will be given the option to inform the study team of non-eligibility by email within 72 hours (see Appendix A). If the 72-hour period elapses with no response, the parent will be considered eligible.

5.2 Consent Process:
Following provider permission, eligible parents/guardians will be approached consecutively by the study team while in clinic or inpatient and offered an opportunity to participate following written informed consent. The study team will offer discussion in a private setting (e.g. consult room) and explain details of the study including study procedures, study risks and benefits and study goals and methods. This will include a discussion of randomization to UC versus PediCARE. The voluntary nature of participation will be highlighted. If, after being introduced to the study and having had the opportunity to ask questions, parent/guardians are willing to participate, they will be asked to sign a written informed consent document.

Dyadic children represent the cohort for analysis, child disease and treatment data will be abstracted from the medical record. Children will experience no direct research contact as a result of participation in the study. Consequently, child assent to participate (medical record abstraction only) will be waived. The justification for this is that parents frequently consider household income/material resource needs to be a sensitive and private topic which they do not wish to share or burden their children with. A requirement to assent children to medical record abstraction will require parental discussion of this topic with children. To this end, parent-participation may be limited were we to require child assent to participation.

Should a child reach the age of majority while enrolled on study, study interventions will continue. Age of majority consent will be waived for these individuals given that patients will experience no direct research contact as a result of participation in the study. Parents/guardians will serve as the survey informants and intervention recipients on behalf of their children, and medical record abstraction does not require patient contact. The justification for this is that parents frequently consider household income/material resource needs to be a sensitive and private topic which they do not wish to share or burden their children with. A requirement to complete age of majority consent would require parental discussion of this topic with children and could limit parent-participation were we to require age of majority consent.

5.3 Registration Process:
All patients, including those enrolled at University of Alabama Birmingham Children's of Alabama (UAB), will be registered in the protocol registration database and assigned a study ID number in the Clinical Trials Management System (CTMS) Oncore.

Registration Process for DFCI:
A member of the study team will confirm eligibility criteria and complete the protocol-specific eligibility checklist.

DF/HCC Standard Operating Procedure for Human Subject Research Titled *Subject Protocol Registration* (SOP #: REGIST-101) must be followed.

Registration Process of UAB:
Eligible patients will be registered on study centrally at the DFCI by the Study Coordinator in real time. Once registration is confirmed, the DFCI study staff facilitating the registration will notify the UAB study staff regarding confirmation of registration. To register a participant, the following documents should be sent to the DFCI Study Coordinator:

- Signed participant consent form
- HIPAA Authorization Form
- Eligibility Checklist

All patients will be randomized to either the PediCARE intervention or Usual Care using the RedCap Database. Each site will be responsible for randomizing their own patients in RedCap.

5.4 Strategies for retention:
We will actively retain families in this study by utilizing ongoing face-to-face study team contact to maintain engagement as well as modest remuneration for ongoing participation. Participants will not be expected to experience any economic burden as part of this study. Enrolled families will receive small ($50 Amazon gift card) remuneration to off-set the cost of their time participating in baseline and follow-up surveys (total value $100 if complete both surveys). Prior non-interventional survey-based studies of HMH by our group have demonstrated excellent retention over time.

Due to the relatively low-participant burden, modest remuneration, and patient-centered nature of the intervention we do not anticipate problems with recruitment and retention.

6.0 Multi-Site Research

6.1 Study Team:
DFCI will serve as the lead site for PediCARE and the single IRB of record for this small, pilot feasibility intervention study. The Study Team at each site will participate in monthly study phone calls to address (a) accrual rate, (b) study eligibility determination issues, (c) data completion rates including conformance with informed consent requirements, (d) intervention fidelity indicators, (e) adverse events, and (f) compliance with data management procedures.

6.2 Collaborating Sites and Role of Sites in Performing Proposed Research:
This study will be conducted at both DFCI and University of Alabama Birmingham Children's of Alabama (UAB). Inclusion of UAB as a collaborating site in this pilot study will leverage patient catchment areas which include both New England and Southern states to recruit a more diverse patient population from the perspective of race, ethnicity, geography and culture—particularly meaningful in the context of poverty as a social determinant of health. Furthermore, pilot feasibility at diverse sites will inform subsequent scalability of our approach for a multi-center RCT intervention study to definitively evaluate the impact of PediCARE on childhood cancer outcomes.

Under the direction of the site PI Dr. Wolfson, UAB will recruit and enroll a total of 12 participants in study. Participants will be randomized to receipt of PediCARE or UC. UAB will maintain a local

patient tracking database, communicate participant enrollment data to DFCI for central administration of randomization and intervention components; administer baseline and follow-up parent surveys; and transmit required source documentation to DFCI for central data monitoring.

7.0 Study Timelines

New patients at each site are continuously identified by study teams. Eligible parents will be approached sequentially by study teams and offered an opportunity to participate following written informed consent. Participants who choose to enroll will be on study for 6-months.

8.0 Study Endpoints

8.1 Feasibility: The primary endpoint of this study is feasibility. Feasibility will be defined as at least 75% consent to randomization and at most 20% attrition per arm. We will assess process barriers to feasibility including recruitment, attrition by arm and parent report of PediCARE component utilization (i.e. food and transportation).

**Study Timeline (Months)**	**Months 1 -6 Year 1**	**Months 7-12 Year 1**	**Months 1-6 Year 2**
Screen and enroll families (6 months)	■	■	
Intervention administration (6 months)	■	■	
Data analysis			■

8.2 HMH and Utilization: The secondary endpoints of this study will be used to generate effect size estimates to support planning of a subsequent RCT. Secondary endpoints will be evaluated comparing recipients of PediCARE versus Usual Care. Secondary outcomes include change in HMH utilizing an HMH score (range 0-4) at 6-months compared to baseline; and proportion of patients experiencing an Emergency Department (ED) visit or Intensive Care Unit (ICU) admission over 6-months.

9.0 Study Procedures

Study Schema

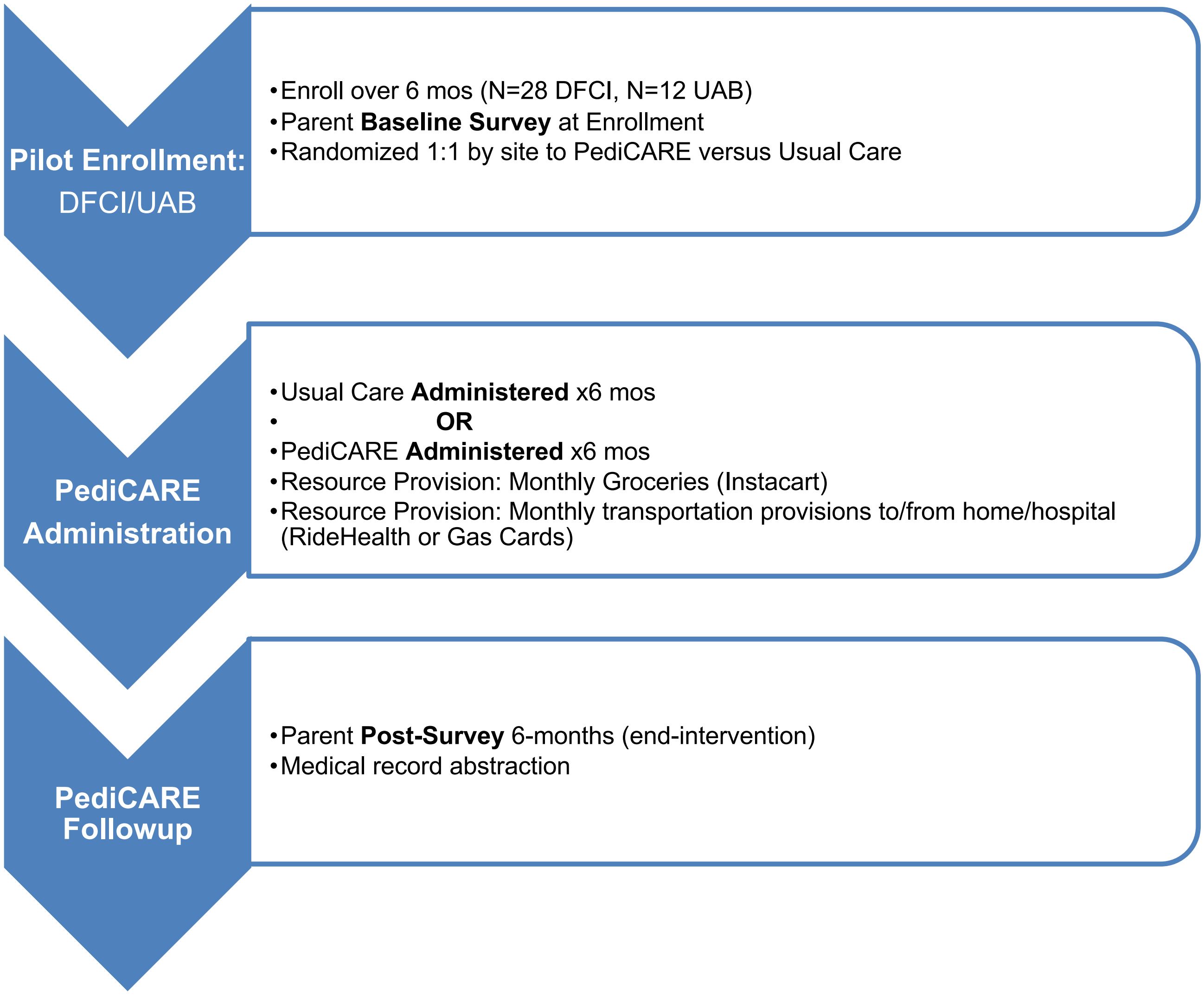

9.1 Overall Study Design

PediCARE is a poverty-targeted intervention utilizing direct resource provision of groceries and transportation. We will pilot PediCARE in a randomized fashion in 40 newly diagnosed families with HMH at DFCI (N=28) and UAB (N=12) to assess feasibility and preliminary change in HMH and care utilization. Human subject participation includes:

1) Parent-completed baseline and post-intervention survey;
2) Randomized receipt of PediCARE versus UC over 6-months;
3) Medical record abstraction of child's chart.

9.1.2 Study Processes:

- Newly diagnosed families continuously screened for study eligibility; eligible families approached to offer consent consecutively (as detailed in section 5.1 Recruitment)
- All parents will complete the baseline HMH survey with the study CRA at time of enrollment. For those parents for whom in-person survey administration is infeasible, baseline surveys will be conducted by telephone, by virtual/video platforms, or by a hard-copy given to the parent to complete on their own and return to the study team.
- Post-survey completion, parents will be randomized to receipt of PediCARE or UC as detailed in Section 9.2. Parents randomized to PediCARE will be introduced to the food and transportation resource provisions by the site CRA (see section 9.3.2).
- Parents will receive the PediCARE intervention or Usual Care (UC) for a total of 6-months.
- Parents will complete a post-intervention survey at 6-months. All parents will be contacted by email or telephone (per family preference) in the weeks prior to the 6-month follow-up survey. Parents will then complete the 6-month follow-up survey with the study CRA. For those parents for whom in-person survey administration is infeasible, follow-up surveys will be conducted by telephone, by virtual/video platforms, or by a hard-copy given to the parent to complete on their own and return to the study team.
- Medical record abstraction performed at 6-months.
  Enrolled parents will receive a total of $100 in Amazon gift cards over the 6-months of study participation ($50 gift card after baseline survey and $50 gift card after post-intervention follow-up survey) to off-set the burden of their time.

9.1.3 Study Measures

HMH Survey Administration: Parents will complete the baseline HMH survey at time of enrollment; parents will complete the abbreviated follow-up HMH survey at intervention completion (6-months). The HMH survey constitutes an abbreviated version of the Oncology EIS survey which has been utilized in two prior studies with high willingness to participate and low participant burden.[10,17] Survey domains include: 1) Sociodemographics; 2) HMH (housing, transportation, utilities, food); 3) Finances; 4) Current resources; 5) Social supports; 5) Resilience; and 6) Anxiety. Survey administration takes approximately 10-15 minutes and may be conducted with an interpreter. Surveys are preferentially administered face-to-face (questions read-aloud by CRA) to overcome health literacy barriers which are of particular pertinence in the target patient population for this study. Surveys may be administered either on an iPad or paper/pencil. For two-parent households, while one parent consents to study participation and serves as the primary survey respondent (e.g. demographics) it is permissible for both parents to contribute information to questions on household material hardship and finances per family preference. All families will be contacted by email or telephone (per family preference) in the week prior to the 6-month follow-up survey to remind them of the upcoming survey time-point. Families will then complete the 6-month follow-up survey with the study CRA either in clinic or inpatient. For those families for whom in-person survey administration is infeasible surveys may be conducted by telephone, virtual platform, or self-completed hardcopy as needed.

Medical Record Abstraction: Child demographic and clinical data will be collected via systematic chart review by trained CRAs at the completion of study receipt (6-months) and entered into a REDCap database. Ten percent of records will be abstracted by a second CRA and discrepancies will be reviewed to clarify abstraction methods and adjudicated by the PI and site PI. Abstracted data elements will include but not be limited to age, diagnosis, cancer risk group or stage, treatment protocol, treatment elements (chemotherapy, surgery, radiation), hospital lengths of stay, emergency department visits and ICU stays during intervention.

9.2 Procedures for Assignment to Study Group:

Enrolled participants will be randomized 1:1 by site to receive either the PediCARE intervention or UC for a duration of 6-months.

Participants will be randomly assigned to PediCARE or UC with a 1:1 allocation by site as per a computer-generated randomization sequence created by the study statistician. Sequences will be embedded in REDCap by informatics personnel. This approach to randomization will allow for real-time randomization of participants. Study personnel will not have access to the sequences. After randomization, participants and study team will be unblinded to group assignment as the intervention is not amenable to blinding.

9.3 Intervention Procedures:

The PediCARE study will pilot direct resource provision (groceries and transportation) to households of children newly diagnosed with cancer with a goal of reducing a concrete measure of poverty, household material hardship (HMH).

9.3.1 Usual Care (UC) Components (Control): UC psychosocial support in pediatric cancer is institution-specific but routinely includes (1) a psychosocial clinician available as part of the cancer care team; (2) social work screening for social and financial needs including such examples as insurance coverage and eligibility for Institutional, community or private cancer foundation supports; (3) social work assistance with applications on a case by case basis for insurance (i.e. Medicaid) and community/institutional/private financial supports; and (4) provision of parking vouchers and/or limited gift cards (e.g. cafeteria and hospital-retail food) when available.

9.3.2 PediCARE Intervention Components: PediCARE includes direct resource provision in two domains: Food and Transportation. Resource provision will be centrally administered by DFCI.

(1) Food: Families will receive a monthly pre-paid allocation of Instacart funds for grocery purchasing and delivery in a dollar amount adapted from the March 2018 U.S. Department of Agriculture's (USDA) Thrifty Food Plan (see Appendix B) for family size (e.g. family of 4 with two toddlers will receive $640; cost range based on family sizes of 2-7 people and capped at 7 people). The Thrifty USDA Food Plan serves as the national standard for a nutritious diet at a minimal cost and is used as the basis for maximum SNAP benefit (e.g. food stamp) allotments, as such it represents a valid bench-mark for household food costs.

- At study entry, the site CRA will assist families randomized to PediCARE in creation of an Instacart account for online grocery purchasing and delivery. Account creation requires

a name, email address, telephone number, home address for grocery delivery, and a credit card, debit card, or gift card

- Families will subsequently be provided a monthly pre-paid allocation of Instacart funds the beginning of each month of study participation. Families will choose and order groceries at their discretion over the course of the study.
- Other logistics: Instacart grocery ordering can be performed on any smartphone or computer with internet access. Any enrolled family without access to either a smartphone or computer will be provided with access to an iPad in clinic or while inpatient for ordering. Groceries can be ordered for same-day or future delivery. Families who live in ZIP codes where Instacart delivery is not available (approximately 15% of the U.S.) will be provided with an equivalent dollar amount grocery gift card to their local grocery store.

(2) Transportation: Families will be provided with the option of either 48 round-trip rides (96 legs) over the course of six months to use as needed each month using RideHealth or (b) gas cards and parking passes to support an estimated 8 clinic/hospital visits per month. The flexibility of choosing which direct transportation support will best facilitate a family's ability to bring their child to clinic or the hospital is based on qualitative parent feedback from the PediCARE pretest.

(a) Transportation will be provided via RideHealth, a company which provides a HIPAA-compliant digital platform to coordinate patient transportation utilizing Uber/Lyft and other commercial ride services, a web-based on-demand transportation platform. This platform allows for HIPAA-compliant account generation using PHI on the back-end, and then links to ride-scheduling with Uber/Lyft or other commercial drivers on the front-end without PHI. Built for healthcare, RideHealth allows ride-authorization for patients between specified sites (i.e. home and hospital/clinic). Rides are booked by the parent, and can be booked in advance (e.g. for a planned clinic visit or hospital stay) or on-demand 24-hours a day via their website or dedicated call center (e.g. for unplanned clinic or Emergency Department visits). RideHealth provides real-time analytics on utilization and performance including timeliness and cost.

- At study entry, the lead-site CRA will create a RideHealth account for families randomized to PediCARE within the central DFCI RideHealth account. Families can view only their own information to facilitate ride requests. Each account will include pre-authorization for 48 round-trip rides (96 legs) over six months.
- The local site CRA will educate families on utilization of the RideHealth patient-facing app to request rides (process is similar to Uber/Lyft app for ride scheduling)
- Ride data including utilization, cost, and time will be provided to the lead site linked to study ID by RideHealth

(b) Families will be provided with gas cards in a dollar amount stratified by distance from hospital based on estimated average frequency of clinic visits/hospitalization and 2018 gas costs. Specifically, families who live <180 miles from the hospital will be provided with $200/month in gas cards; those who live >=180 miles from the hospital will be provided with $300/month in gas cards. Families will receive 8 parking passes per month at sites where families pay for parking.

PediCARE will provide both groceries and transportation support to all intervention recipients for the duration of the study. Pilot families will be randomized 1:1 at each site to receive the

PediCARE intervention or UC for 6-months which represents the most intensive phase of treatment for children with leukemia and neuroblastoma and covers the duration of chemotherapy treatment for most solid tumor and lymphoma diagnoses.

10.0 Data Management and Confidentiality

10.1 HIPAA Authorization and Waivers: The following protected health information (PHI) is needed to carry out the study: contact information (names, addresses, phone numbers, and email address) necessary to identify subjects, ask for consent, and ensure follow-up contact with subjects throughout the study; medical record numbers are needed to abstract medical information; child date of birth and diagnosis, necessary to calculate the child's age and time passed since diagnosis; parent date of birth, necessary to calculate parent age. PHI data will be stored in a HIPAA compliant REDCap database accessible only to the study team, all of whom have HIPAA Certification and have completed the training mandated by the DFCI Institutional Review Board.

A HIPAA Waiver of Authorization will be requested to enable thorough screening for eligibility. This process involves no more than a minimal risk to subjects' privacy because identifiers will be collected in a secure way and destroyed after the study is closed. In addition, the protected health information collected will not be reused or disclosed to any other person or entity other than the research team, except as required by law. Because we aim to enroll participants as close to the time of cancer diagnosis as possible, we have a relatively narrow window in which to identify eligible participants. Without such a waiver, we would have to rely solely on provider-generated referrals or patient and parent self-referral, which would add an undue burden to oncology providers and potentially lead to such slow participant accrual that the research would be rendered infeasible. Alternatively, requesting consent from each potential study participant prior to review of preliminary eligibility would result in an unnecessary burden for families.

10.2 Data Management: All information collected for research purposes will be de-identified prior to analysis.

- Identifying information will include data (names, addresses, phone numbers, emails) necessary to identify subjects initially to ask for consent as well as to contact for follow-up survey administration if necessary; child's demographic and disease information.
- The data will be coded, databases secured with a password, and contact information and medical record numbers will be kept in a separate database accessible only to the Principal Investigator and local RA who have both undergone HIPAA Certification and the training mandated by the IRB. Study databases will not contain identifying information, and will be linked to identifying information by a unique study ID.
- A separate password-protected key file containing the link between identifying information and research data will be maintained by the PI behind the DF/HCC firewall. We have extensive experience in maintaining confidentiality utilizing these methods.

10.3 Use of Data for Intervention Administration for Parents receiving PediCARE:

- Per the Business Agreement, parent contact information (specifically email address and home address) with study ID number will be utilized for creation of a RideHealth account which provides HIPAA compliant privacy protections.

- Parents will create their own Instacart account which will not be linked to study ID and to which research team will not have access. Parent email address will be utilized to send an Instacart gift card for each month of the intervention.

11.0 Statistical Considerations

Accrual: Annually, 420 new patients receive chemotherapy at DFCI and 120 at UAB. Based on prior institutional HMH data, we estimate 20% will be eligible to participate at DFCI (N=84) and 30% will be eligible to participate at UAB (N=36). We estimate a 75% consent rate for a total possible annual sample size of N=90 patients (63 DFCI, 27 UAB). Forty patients will be accrued over 6-months at DFCI (n=28) and UAB (n=12). Patients will be randomized 1:1 by site to PediCARE versus UC.

Analysis Plan: Sample demographic, disease, treatment and utilization variables from the survey instruments and medical chart will be described by summary statistics.

The rate of consent to randomized intervention and the 6-month completion will be estimated along with a 90% confidence interval (CI). Feasibility will be established if the upper bound of the CI contains 75% and 80% for consent and follow-up respectively. With an estimated 60 eligible patients at 6 months, at least 39 (65%; 90% CI 53.6-75.2%) need to enroll for the upper bound of the 90% CI contain 75%. With an estimated 40 randomized patients, at least 28 (70%; 90% CI 56.0-81.7%) need to have complete 6-month data to have the upper bound of the 90% CI contain 80%. The 6-month completion rate will also be estimated along with a 90% CI by randomized arm (PediCARE and UC).

For each family, the difference between 6-month and baseline HMH scores will be categorized as improved (change score <0) or not improved (change score ≥0). For each arm, the proportion of families with improved HMH at the completion of the intervention (6-months) will be estimated along with a 90% exact CI. Based on prior data, we assume a 30% resolution in HMH in UC, thus the 90% CI will be within ±24.9%. For each arm, the per patient number of ED visits and ICU stays will be summarized (median, range). The proportion of patients with at least one ED and/or ICU visit will be estimated for each arm, as well as the difference between arms, along with a 90% confidence interval. Additionally, the rate of HMH at the completion of the intervention pilot (6-months) along with a 90% exact confidence will be estimated for UC and PediCARE. For the PediCARE arm, the cost per patient, study team contact hours, and proportion with successful receipt of all intervention components will be described overall and by site.

12.0 Data Monitoring Provisions to Ensure the Safety of Subjects

See Appendix E, Section 4 for Data Safety Monitoring Plan.

13.0 Risks to Subjects

Potential Risks to Subjects: Risks for this pilot resource provision intervention are detailed below. Participants in this study include children (medical record abstraction only) with parent/guardians as survey informants and intervention recipients on their behalf. Children will have medical record

abstraction only and experience no study contact. All risks of study involvement are expected to be minimal.

1. Physical risks: There are no physical risks to subjects in the proposed study.

2. Psychological risks: Parents/guardians will serve as survey informants and intervention recipients. Surveys deal with the sensitive matter of family finances and concrete resource needs. Family finances may be a source of stress for parents/guardians. The emotional risk of anxiety as a result of answering questions about financial hardship is expected to be minimal based on data from our prior research. Specifically, prior work on this topic has revealed that parents are eager to participate and do not report significant stress or anxiety as a result of participation; furthermore, parents participating in prior studies have identified an opportunity to speak about financial stresses as being beneficial to them. Participants may decide at any time and for any reason not to participate in the proposed study. Receipt of the intervention will include receipt of prepaid groceries and prepaid transportation between home and hospital. We expect minimal psychological risk as a result of receiving such resource provision.

3. Privacy risks: There is a risk of privacy violation or loss of confidentiality; however, this is anticipated to be minimal with appropriate protections in place (see Protection Against Risks below).

4. Burden to families due to participation time: Burden is expected to be minimal. Children will have no direct study interaction and will not experience burden from participation. Parents will complete a baseline survey (approximately 15 minutes) and a post-intervention survey (approximately 10 minutes). These surveys have been administered over 300 times in other HMH studies with minimal parent burden.

5. Risk resulting from randomization: As a result of randomization, participants may not receive the intervention that ends up being more efficacious. For example, receiving UC (control arm) with site social workers and psychosocial clinicians addressing resource needs may result in persistent or worsened HMH. However, it is expected if persistent HMH is identified in the course of UC such basic resource needs will be addressed by the psychosocial team as usual which includes a robust assortment of standard supports and interventions. Conversely, being exposed to the PediCARE intervention may result in receipt of an intervention that has no ultimate impact on HMH. The risks to receipt of pre-paid groceries and transportation are expected to be minimal.

Alternatives: There are no alternatives to this study, subjects may choose not to enroll in the PediCARE pilot study and will receive usual psychosocial support care per institutional standard. Subjects will be counseled during the consent process that participation is entirely voluntary and that a decision not to participate will in no way affect the care their child receives. Participants will have the choice to exit the study at any time, should they desire to do so.

14.0 Potential Benefits to Subjects

Potential benefits of the research to research participants and others.
Participants in this study have the potential for direct benefit in the form of poverty-reduction due to either increased awareness of UC resources created by study participation, or due to randomized receipt of PediCARE. The primary goal of the PediCARE intervention is to reduce household material hardship (HMH), a concrete measure of poverty which includes food and transportation insecurity. All participants enrolled in the PediCARE study will have screened positive for the presence of HMH at diagnosis. This screening process is part of UC at both DFCI and UAB, however follow-up beyond screening is not systematic and eligibility for existing community and governmental resources is not universal. Participants enrolled and randomized to receipt of the PediCARE intervention will be provided with pre-paid groceries and transportation for the duration of their study involvement. Participants in receipt of the PediCARE intervention may experience a reduction in HMH which we believe would be of benefit to the household.

15.0 Sharing of Results with Subjects

The proposed pilot feasibility study will generate data on approximately 40 pediatric oncology families with newly diagnosed cancer treated at two major cancer centers in the United States. Aggregate analyses of de-identified data will be published in peer reviewed journals within 1 year of study completion. Published data will be free of any identifiers that would permit linkages to individual subjects. We will present our intervention refinement process, our qualitative family data and our pilot feasibility data at professional meetings/conferences such as ASCO and ASH within 1 year of study completion. We will present our findings to patient advocacy organizations such as the Pediatric Parent and Family Council and the Family Reach Foundation, and disseminate to reputable patient resource sites (i.e. NIH/NCI resources for patients).

16.0 Study Evaluations Table

	Intervention Group	Usual Care Group
**Screening (HIPAA Waiver Requested)**		
Diagnosis type/date	X*	X*
Date of Birth	X*	X*
Primary oncology team	X*	X*
Planned chemotherapy regimen	X*	X*
Primary language	X*	X*
Planned location of treatment	X*	X*
16-001 enrollment (DFCI only)	X*	X*
Sociodemographics	X	X
**Following Informed Consent**		
Baseline parent survey	X	X
Randomization	X	X
Remuneration		X
**Each month on study**		
Resource provision (PediCARE)	X	
Usual Care		X
**End of study**		
Follow-up parent survey	X	X
Remuneration	X	X
Clinical and treatment characteristics abstraction	X*	X*
Health Care Utilization Data Abstraction	X*	X*

* This item will be extracted from the child's medical record

17.0 References

1. Macartney S. Child Poverty in the United States 2009 and 2010: Selected Race Groups and Hispanic Origin American Community Survey Briefs. US Census Bureau, 2011. Available at: http://www.census.gov/prod/2011pubs/acsbr10-05.pdf. 2011 ed.
2. Gupta S, Wilejto M, Pole JD, Guttmann A, Sung L. Low socioeconomic status is associated with worse survival in children with cancer: a systematic review. *PloS one.* 2014;9(2):e89482.
3. Petridou ET, Sergentanis TN, Perlepe C, et al. Socioeconomic disparities in survival from childhood leukemia in the United States and globally: a meta-analysis. *Annals of oncology : official journal of the European Society for Medical Oncology / ESMO.* 2015;26(3):589-597.
4. Bona K, Blonquist TM, Neuberg DS, Silverman LB, Wolfe J. Impact of Socioeconomic Status on Timing of Relapse and Overall Survival for Children Treated on Dana-Farber Cancer Institute ALL Consortium Protocols (2000-2010). *Pediatric blood & cancer.* 2016;63(6):1012-1018.
5. Meyers A, Cutts D, Frank DA, et al. Subsidized housing and children's nutritional status: data from a multisite surveillance study. *Archives of pediatrics & adolescent medicine.* 2005;159(6):551-556.
6. Cook JT, Frank DA, Berkowitz C, et al. Food insecurity is associated with adverse health outcomes among human infants and toddlers. *The Journal of nutrition.* 2004;134(6):1432-1438.
7. Frank DA, Neault NB, Skalicky A, et al. Heat or eat: the Low Income Home Energy Assistance Program and nutritional and health risks among children less than 3 years of age. *Pediatrics.* 2006;118(5):e1293-1302.
8. Black MM, Cutts DB, Frank DA, et al. Special Supplemental Nutrition Program for Women, Infants, and Children participation and infants' growth and health: a multisite surveillance study. *Pediatrics.* 2004;114(1):169-176.
9. Garg A, Toy S, Tripodis Y, Silverstein M, Freeman E. Addressing social determinants of health at well child care visits: a cluster RCT. *Pediatrics.* 2015;135(2):e296-304.
10. Bona K, London WB, Guo D, Frank DA, Wolfe J. Trajectory of Material Hardship and Income Poverty in Families of Children Undergoing Chemotherapy: A Prospective Cohort Study. *Pediatric blood & cancer.* 2016;63(1):105-111.
11. Berkowitz SA, Hulberg AC, Standish S, Reznor G, Atlas SJ. Addressing Unmet Basic Resource Needs as Part of Chronic Cardiometabolic Disease Management. *JAMA Intern Med.* 2016.
12. Sege R, Preer G, Morton SJ, et al. Medical-Legal Strategies to Improve Infant Health Care: A Randomized Trial. *Pediatrics.* 2015;136(1):97-106.
13. O'Leary M, Krailo M, Anderson JR, Reaman GH, Children's Oncology G. Progress in childhood cancer: 50 years of research collaboration, a report from the Children's Oncology Group. *Seminars in oncology.* 2008;35(5):484-493.
14. Ward E, DeSantis C, Robbins A, Kohler B, Jemal A. Childhood and adolescent cancer statistics, 2014. *CA: a cancer journal for clinicians.* 2014;64(2):83-103.
15. Bhatia S, Landier W, Hageman L, et al. 6MP adherence in a multiracial cohort of children with acute lymphoblastic leukemia: a Children's Oncology Group study. *Blood.* 2014;124(15):2345-2353.
16. Bona K, London WB, Guo D, Abel G, Lehmann L, Wolfe J. Prevalence and impact of financial hardship among New England pediatric stem cell transplantation families. *Biol Blood Marrow Transplant.* 2015;21(2):312-318.
17. Gupta S, Sutradhar R, Guttmann A, Sung L, Pole JD. Socioeconomic status and event free survival in pediatric acute lymphoblastic leukemia: a population-based cohort study. *Leukemia research.* 2014;38(12):1407-1412.
18. Warner EL, Kirchhoff AC, Nam GE, Fluchel M. Financial Burden of Pediatric Cancer for Patients and Their Families. *Journal of oncology practice / American Society of Clinical Oncology.* 2014.
19. Kids Count Data Center. 2014. http://datacenter.kidscount.org.

20. Newacheck P, Jameson WJ, Halfon N. Health status and income: the impact of poverty on child health. *The Journal of school health.* 1994;64(6):229-233.
21. Singh G. Child Mortality in the United States, 1935-2007: Large Racial and Socioeconomic Disparities Have Persisted Over Time. Accessed December 17, 2012.
22. Summary Health Statistics for U.S. Children: National Health Interview Survey, 2010. 2011. Accessed December 18, 2012.
23. Lightfoot TJ, Johnston WT, Simpson J, et al. Survival from childhood acute lymphoblastic leukaemia: the impact of social inequality in the United Kingdom. *Eur J Cancer.* 2012;48(2):263-269.
24. Cook JT, Frank DA, Casey PH, et al. A brief indicator of household energy security: associations with food security, child health, and child development in US infants and toddlers. *Pediatrics.* 2008;122(4):e867-875.
25. Yoo JP, Slack KS, Holl JL. Material hardship and the physical health of school-aged children in low-income households. *American journal of public health.* 2009;99(5):829-836.
26. Coleman-Jensen A NM, Andrews M and Carlson S. Household Food Security in the United States in 2013. *US Department of Agricultrue, Economic Research Service.* 2013;ERR 173.
27. Coleman-Jensen A MPR, Christian A. Gregory, and Anita Singh. Household Food Security in the United States in 2016, ERR-237. In: U.S. Department of Agriculture ERS, ed2017.
28. Joyce Kea. Household Hardships, Public Programs, and Their Associations with the Health and Development of Very Young Children: Insights from Children's HealthWatch. *Journal of Applied Research on Children: Informing Policy for Children at Risk.* 2012;3(1).
29. Edin K MB, James Mabli, Jim Ohls, Julie Worthington, Sara Greene, Nicholas Redel, Swetha Sridharan. "SNAP Food Security In-Depth Interview Study: Final Report". In: U.S. Department of Agriculture FaNS, ed. Alexandria, VA: Office of Research and Analysis March 2013.
30. Board TR, National Academies of Sciences E, Medicine. *Cost-Benefit Analysis of Providing Non-Emergency Medical Transportation.* Washington, DC: The National Academies Press; 2005.
31. Syed ST, Gerber BS, Sharp LK. Traveling towards disease: transportation barriers to health care access. *J Community Health.* 2013;38(5):976-993.
32. Powers BW, Rinefort S, Jain SH. Nonemergency Medical Transportation: Delivering Care in the Era of Lyft and Uber. *JAMA.* 2016;316(9):921-922.
33. Shier G, Ginsburg M, Howell J, Volland P, Golden R. Strong social support services, such as transportation and help for caregivers, can lead to lower health care use and costs. *Health affairs (Project Hope).* 2013;32(3):544-551.
34. Wolfson J, Sun CL, Wyatt L, Stock W, Bhatia S. Impact of treatment site on disparities in outcome among adolescent and young adults with Hodgkin lymphoma. *Leukemia.* 2017;31(6):1450-1453.
35. Wolfson J, Sun CL, Wyatt L, Stock W, Bhatia S. Adolescents and Young Adults with Acute Lymphoblastic Leukemia and Acute Myeloid Leukemia: Impact of Care at Specialized Cancer Centers on Survival Outcome. *Cancer Epidemiol Biomarkers Prev.* 2017;26(3):312-320.
36. Wolfson J, Sun CL, Kang T, Wyatt L, D'Appuzzo M, Bhatia S. Impact of treatment site in adolescents and young adults with central nervous system tumors. *Journal of the National Cancer Institute.* 2014;106(8):dju166.
37. Bona K BT, Neuberg DS, Silverman LB and Wolfe J. . Impact of Socioeconomic Status on Timing of Relapse and Overall Survival for Children Treated on Dana-Farber Cancer Institute ALL Consortium Protocols (2000-2010). *Pediatric Blood Cancer.* 2016.
38. Kessler RC, Andrews G, Colpe LJ, et al. Short screening scales to monitor population prevalences and trends in non-specific psychological distress. *Psychological medicine.* 2002;32(6):959-976.
39. United States Department of Agriculture, Economic Research Service. *U.S. Household Food Security Survey Module: Six-Item Short Form.* September 2012. Available at: http://www.ers.usda.gov/briefing/foodsecurity/surveytools/hh2008.pdf. . 2008.
40. Children's HealthWatch Survey. 2010.

41. Wolfe J, Grier HE, Klar N, et al. Symptoms and suffering at the end of life in children with cancer. *N Engl J Med.* 2000;342(5):326-333.
42. Prawitz A. In Charge Financial Distress/Financial Well-Being Scale: Development, Administration, and Score Interpretation. *Financial Counseling and Planning.* 2006;17(1):34-50.
43. Official USDA Food Plans: Cost of Food at Home at Four Levels, U.S. Average, July 2017. In: Agriculture USDo, ed.

44. Carlson A, Lino, M., & Fungwe, T. . The Low-Cost, Moderate-Cost, and Liberal Food Plans, 2007 (CNPP-20). . In: U.S. Department of Agriculture Cf, Promotion. NPa, eds2007.

45. https://www.cnpp.usda.gov/sites/default/files/CostofFoodMar2018.pdf. Accessed April 23, 2018.
46. Hager ER, Quigg AM, Black MM, et al. Development and validity of a 2-item screen to identify families at risk for food insecurity. *Pediatrics.* 2010;126(1):e26-32.
47. Blumberg SJ, Bialostosky K, Hamilton WL, Briefel RR. The effectiveness of a short form of the Household Food Security Scale. *American journal of public health.* 1999;89(8):1231-1234.
48. Moustakas C. *Phenomenological Research Methods.* Sage; 1994.
49. SM L. Hermeneutic phenomenology and phenomenology: A comparison of historical and methodological considerations. *International Journal of Qualitative Methods.* 2003;2(3):1-29.
50. Cohen MZ KDaRS. How to analyze data. *Hermeneutic phenomenological research: A practical guide for nurse researchers*: Sage; 2000:71-83.

18.0 Appendices

Appendix A: Grocery Allocation Household Size Table
Appendix B: Parent surveys (baseline and end-intervention)
Appendix C: Instacart information sheet
Appendix D: Data Safety Monitoring Plan
Appendix E: Ride Health information sheet

Appendix A. Grocery Allocation Household Size Table

Household Size	Monthly Provision
2	$360
3	$510
4	$640
5	$760
6	$920
>=7	$1000

Appendix B. Parent Surveys (Baseline and Post-Intervention)

Study ID number: ____________

PediCARE Survey

Baseline Survey (T0)

Nearly 1 in 3 families experience problems with money or financial stress during their child's cancer treatment. This may include lost income from work, difficulty paying the rent or mortgage, keeping the electricity or heat on, or putting food on the table. Financial stresses or problems with money can affect both adults and children in many ways. One of our long-term goals is to find ways to decrease the financial impact of treatment on each family, and to make sure that financial stress does not impact a child's experience during treatment. Information from families like yours will help us achieve this goal.

This survey has multiple-choice questions and will take about 15 minutes to complete. The survey is divided into sections that cover topics about you and your family, housing, utilities (heat/electricity), food, finances, home environment, stress and neighborhood. We understand that some of these questions may feel personal and difficult to answer. You may skip any questions and even stop completing the survey at any time.

Survey responses will be kept confidential. Your answers will not be shared with your treatment team. Survey data will not be placed in your child's medical record.

Before we analyze the survey data for research they will be de-identified, which means that no information that would permit identification of your family or child will be shared.

A. Demographics

The following questions ask you a bit about yourself and your family.

About You:

1. What is your relationship to the patient?
   a. Mother
   b. Father
   c. Legal Guardian (please specify relationship): ____________________________
   d. Other Guardian (please specify): _____________________________________

2. How old are you?
   a. Age in years: _____
   b. Prefer not to answer

3. What is the primary language you speak at home?
   a. English
   b. Spanish
   c. Other, please specify: ___________________

4. In what language do you prefer to have medical conversations about your child?
   a. English
   b. Spanish
   c. Other: __________

5. How confident are you filling out medical forms by yourself?
   a. Not at all
   b. A little bit
   c. Somewhat
   d. Quite a bit
   c. Extremely

6. Please describe your marital status:
   a. Single
   b. Married or living together with partner
   c. Widowed
   d. Separated or divorced
   e. Other: ______

7. What is the highest level of education <u>you</u> received? *Please choose the best response:*
   a. Grade school/elementary school (Grades 1-8)
   b. Started high school but didn't graduate (Grades 9-12)
   c. High school diploma or GED
   d. Trade/technical/vocational training
   e. Some college
   f. College graduate
   g. Postgraduate (Master's or Doctorate)

8. If your child has another caregiver/parent, what is the highest level of education he/she received? *Please choose the best response:*
   a. Not applicable
   b. Grade school/elementary school (Grades 1-8)
   c. Started high school but didn't graduate (Grades 9-12)
   d. High school diploma or GED
   e. Trade/technical/vocational training after high school
   f. Some college
   g. College graduate
   h. Postgraduate (Master's or Doctorate)

About your child:

9. What was your child's health insurance <u>just before his/her diagnosis</u>:
   a. Private insurance
   b. Medicaid or Children's Health Insurance Program (CHIP) (public insurance)
   c. Private insurance <u>plus</u> Medicaid/CHIP
   d. Uninsured/Self-pay
   e. Other (please specify e.g. Military): ____________

10. In general, how would you rate your child's health <u>prior to this diagnosis of cancer</u>?
   a. Excellent
   b. Very good
   c. Good
   d. Fair
   e. Poor

11. Is your child Hispanic, Latino/a, or Spanish origin?
   a. Yes, Hispanic, Latino/a, or Spanish origin
   b. No, not of Hispanic, Latino/a, or Spanish origin

12. What is your child's race? *Please choose **all that apply**:*
    a. American Indian/Alaskan Native
    b. Asian
    c. Black or African-American
    d. Native Hawaiian or Other Pacific Islander
    e. White

B. Housing and Transportation

The following questions ask about the primary home in which your child lives. Please answer these questions ***about the home in which your child lived before his/her cancer diagnosis.***

1. What is your current zip code? ____________

2. Please tell me what type of housing you and your child live in (choose the best response):
   a. Apartment
   b. House/Townhouse/Condo
   c. Mobile home/trailer
   d. Room/rented room
   e. Shelter or transitional living situation
   f. No steady place to sleep at night (e.g. temporarily staying with others, in a hotel, on the street, in a car, in a park, etc.)
   g. Other (please specify): ______________

3. Do you think you are at risk of becoming homeless?
   a. Yes
   b. No

4. Before your child's diagnosis, how many people (including yourself and your child) were living in your home?
   __________ Number of people
   __________ Of these people, how many are children (ages 0-17 years old)?
   __________ Of these people, how many are adults (ages >=18 years old)?
   __________ Of the adults, how many bring income into the household?

3. How many bedrooms are in your home?
   __________# bedrooms

4. How many places have you and your child lived in the past year?
   __________# of places

5. In the 6 months before your child's diagnosis, was there a time when you were not able to pay the rent or mortgage on time because of financial difficulties?
   a. Yes
   b. No

6. In the last 6 months, have you and your child had to temporarily live with other people because of financial difficulties?
   a. Yes
   b. No

7. How will you get to the hospital or clinic for appointments? *Please choose all that apply.*
   a. Own car
   b. Rides from Others
   c. Public (bus, subway, train)
   d. Uber, Lyft, taxicab etc
   e. Medicaid transportation benefit
   f. Not Sure/Don't know

8. In the last 6 months before your child's diagnosis, has lack of reliable transportation kept you or anyone in your family from medical appointments, meetings, work or getting things needed for daily living?
   a. Yes
   b. No

C. Utilities

The following questions ask about heat and electricity in your home. Please answer these questions thinking about the 6 months ***before your child was diagnosed with cancer.***

1. During the past 6 months, has the gas/electric/oil company sent you a letter threatening to shut off the gas/electricity/oil to the house for not paying bills?
   a. Yes
   b. No
   c. Not applicable, I don't pay for gas/electric/oil

2. During the past 6 months, has the gas/electric/oil company shut off electricity or refused to deliver oil/gas for not paying bills?
   a. Yes
   b. No
   c. Not applicable, I don't pay for gas/electric/oil

3. During the past 6 months, were there any days that your home was not heated/cooled because you couldn't pay the bills?
   a. Yes
   b. No
   c. Not applicable, I don't pay for heat or electricity

4. During the past 6 months, have you had to use a cooking stove to heat your home because you couldn't pay the bills?
   a. Yes
   b. No
   c. Not applicable, I don't pay for heat or electricity

5. During the past 6 months, did you have service disconnected on your telephone (including cell phones) because you couldn't pay the bill?
   a. Yes
   b. No
   c. Not applicable, I don't pay for telephone

D. Food

*The next set of questions are about the food eaten in your household in the last 6 months, and whether you were able to afford the food you need. Please answer these questions thinking about **the 6 months before your child was diagnosed with cancer.***

I'm going to read you two statements that other people have made about their food situation. For each statement, please tell me whether the statement was often true, sometimes true, or never true for your household in the last 6 months.

1. The first statement is, "We worried whether our food would run out before we got money to buy more." Was that often, sometimes, or never true for your household in the last 6 months?
   a. Often true
   b. Sometimes true
   c. Never true
   d. Don't know or prefer not to answer

2. "The food that we bought just didn't last, and we didn't have money to get more." Was that often, sometimes, or never true for your household in the last 6 months?
   a. Often true
   b. Sometimes true
   c. Never true
   d. Don't know or prefer not to answer

E. Finances

The next set of questions are about the resources supporting your child. Please answer these questions thinking about your household ***before your child's diagnosis.***

1. Please provide your best estimate of your total combined household income (i.e. the amount your family would report on your taxes). This should include income from all sources: wages, child support payments, rent from properties, social security, disability and/or veteran's benefits, unemployment benefits, workman's compensation, and so on:
    a. $ ___________
    b. Don't know or prefer not to answer

2. Think about the past 6 months. Would you say that each month you generally ended up with:
    a. More than enough money left over
    b. Some money left over
    c. Just enough to make ends meet
    d. Almost enough to make ends meet
    e. Not enough to make ends meet

3. If you lost all of your current sources of income (for example, your paycheck, Social Security or pension, public assistance) and had to live off of your savings, how long could you continue to live at your current address and standard of living?
    a. Less than 1 month
    b. 1-2 months
    c. 3-6 months
    d. 7-12 months
    e. More than 1 year
    f. Don't know or prefer not to answer

F. Current Resources

*The next set of questions are about the resources supporting your child. Please answer these questions thinking about your household **before your child's diagnosis.***

1. Many families find financial support from existing resources. Please tell us if your family was receiving any of the following supports before your child's diagnosis?

a. Medicaid health insurance for your child
a. Yes
b. No
c. I don't know

b. Food stamps (i.e. SNAP)
a. Yes
b. No
c. I don't know

c. Free school lunch
a. Yes
b. No
c. I don't know
d. Does not apply (my child/children is/are not in school)

d. Food from a food pantry (sometimes called a food bank or soup kitchen)
a. Yes
b. No
c. I don't know

e. Energy assistance (discounted gas/electric bills such as LIHEAP or utility discounts)
a. Yes
b. No
c. I don't know

f. Disability benefits (i.e. SSI, Supplemental Security Income)
a. Yes
b. No
c. I don't know

g. Low-income or subsidized housing (sometimes called Section 8 housing)
a. Yes
b. No
c. I don't know

G. Social Support

People often look to others for companionship, assistance, or other types of support. How often is each of the following kinds of support available to you if you need it? Choose one number from each line.

How often is someone available:	None of the time	A little of the time	Some of the time	Most of the time	All of the time
a) to help you if you were confined to bed?	1	2	3	4	5
b) to take you to the doctor if you need it?	1	2	3	4	5
c) to prepare your meals if you are unable to do it yourself?	1	2	3	4	5
d) to help with daily chores if you were sick?	1	2	3	4	5
e) to have a good time with?	1	2	3	4	5
f) to turn to for suggestions about how to deal with a personal problem?	1	2	3	4	5
g) who understands your problems?	1	2	3	4	5
h) to love and make you feel wanted?	1	2	3	4	5

Any additional comments:

____

____

H. Resilience

The following questions ask about how you handle difficult situations. For each question, please choose the option that best indicates how much you agree with the statement as it applies to you on average, over the last month.

	Not true at all	Rarely true	Sometimes true	Often true	True nearly all of the time
1. I am able to adapt when changes occur.	1	2	3	4	5
2. I can deal with whatever comes my way.	1	2	3	4	5
3. I try to see the humorous side of things when I am faced with problems.	1	2	3	4	5
4. Having to cope with stress can make me stronger.	1	2	3	4	5
5. I tend to bounce back after illness, injury, or other hardships.	1	2	3	4	5
6. I believe I can achieve my goals, even if there are obstacles.	1	2	3	4	5
7. Under pressure, I stay focused and think clearly.	1	2	3	4	5
8. I am not easily discouraged by failure.	1	2	3	4	5
9. I think of myself as a strong person when dealing with life's challenges and difficulties.	1	2	3	4	5
10. I am able to handle unpleasant or painful feelings like sadness, fear, and anger.	1	2	3	4	5

Any additional comments:

I. Parent Well-Being

The following questions ask about your health and well-being.

1. In general, would you say your health is:
    a. Excellent
    b. Very good
    c. Good
    d. Fair
    e. Poor

2. For how long (if at all) has your health limited you in each of the following activities?

	Limited for more than 3 months	Limited for 3 months or less	Not limited at all
The kinds or amounts of **vigorous** activities you can do, like lifting heavy objects, running or participating in strenuous sports	1	2	3
The kinds or amounts of **moderate** activities you can do, like moving a table, carrying groceries, or bowling	1	2	3
Walking uphill or climbing a few flights of stairs	1	2	3
Bending, lifting, or stooping	1	2	3
Walking one block	1	2	3
Eating, dressing, bathing, or using the toilet	1	2	3

Any additional comments:

3. How much bodily pain have you had during the past 4 weeks?
    a. None
    b. Very mild
    c. Mild
    d. Moderate
    e. Severe
    f. Very severe

4. Does your health keep you from working at a job, doing work around the house, or going to school?
    a. Yes, for more than 3 months
    b. Yes, for 3 months or less
    c. No

5. Have you been unable to do certain kinds or amounts of work, housework, or schoolwork because of your health?
    a. Yes, for more than 3 months
    b. Yes, for 3 months or less
    c. No

6. For each of the following questions, please choose the answer that comes closest to the way you have been feeling during the past month:

	**None of the time**	**A little of the time**	**Some of the time**	**Most of the time**	**All of the time**
How much of the time has your **health limited your social activities** (like visiting with friends or close relatives)?	1	2	3	4	5
How much of the time have you been **a very nervous person**?	1	2	3	4	5
How much of the time have you felt **calm and peaceful?**	1	2	3	4	5
How much of the time have you felt **downhearted and blue?**	1	2	3	4	5
How much of the time have you been **a happy person?**	1	2	3	4	5
How often have you felt so **down in the dumps that nothing could cheer you up?**	1	2	3	4	5

Any additional comments:

____

____

7. For the following statements, please choose the option that best indicates how true or false they are for you, over the past month:

	Definitely True	Mostly True	Not Sure	Mostly False	Definitely False
I am somewhat ill	1	2	3	4	5
I am as healthy as anybody I know	1	2	3	4	5
My health is excellent	1	2	3	4	5
I have been feeling bad lately	1	2	3	4	5

Any additional comments:

____

____

8. Over the last 2 weeks, how often have you been bothered by the following problems?

	Not at all	Several Days	More than half the days	Nearly Every Day
Feeling nervous, anxious or on edge	○	○	○	○
Not being able to stop or control worrying	○	○	○	○
Worrying too much about different things	○	○	○	○
Trouble relaxing	○	○	○	○
Being so restless that it is hard to sit still	○	○	○	○
Becoming easily annoyed or irritable	○	○	○	○
Feeling afraid as if something awful might happen	○	○	○	○

Any additional comments:

J. Parent-Patient Healthcare Activation

The following questions ask about your levels of knowledge, skills, and confidence in managing your child's health. For each question, please choose the option that best indicates how much you agree with the statement as it applies to you on average.

	Strongly Disagree	Disagree	Agree	Strongly Agree
1. **When all is said and done, I am the person who is responsible for taking care of my child's health**	1	2	3	4
2. **Taking an active role in my child's health care is the most important thing that affects his/her health**	1	2	3	4
3. **I am confident I can help prevent or reduce problems associated with my child's health**	1	2	3	4
4. **I know what each of my child's immunizations are for**	1	2	3	4
5. **I am confident that I can tell when I need to go get medical care and when I can handle my child's health problem myself**	1	2	3	4
6. **I am confident I can tell a doctor concerns I have about my child's health, even when he or she does not ask**	1	2	3	4
7. **I am confident that I can follow through on medical treatments I need to do for my child at home**	1	2	3	4
8. **I understand my child's health problems and what causes them**	1	2	3	4
9. **I know what treatments are available for my child's health**	1	2	3	4
10. **I have been able to help my child maintain (keep up with) recommended changes like eating right or exercising**	1	2	3	4
11. **I know how to prevent problems with my child's health**	1	2	3	4

12. **I am confident I can figure out solutions when new situations arise with my child's health**	1	2	3	4
13. **I am confident I can help my child maintain changes, like eating right and exercise, even during times of stress**	1	2	3	4

Any additional comments:

We have come to the end of the questionnaire.

To be completed by administering research staff.

Today's date (month/day/year): __ __ / __ __ / __ __ __ __

Site at which survey administered:

a. DFCI
b. UAB

Name of staff member administering survey (Last, First): ______________________

Survey administered:

a. English, US
b. Spanish, US

Interpreter used:

a. Yes (specify language): _______________
b. No

Local Site Tracking Information

For internal site use only.

To be completed by administering research staff for local use and patient tracking.
This page should be removed for survey before scanning survey as source document.

Family's preferred contact information for reminders prior to follow-up surveys:

a. Phone: ____________________
b. Email: ____________________
c. Both

Study ID number: ____________

PediCARE Follow-up Survey

Follow-up Survey (T1)

Earlier studies by our group have shown that many families experience financial stress during their child's cancer treatment, regardless of their household income or socioeconomic status. Financial stress may include lost income from work, difficulty paying the rent or mortgage, keeping the electricity or heat on, or putting food on the table. One of our long-term goals is to find ways to decrease the financial impact of treatment on each family, and to make sure that financial stress does not impact a child's experience during treatment.

You previously completed a baseline survey on this topic. This follow-up survey is intended to help us understand how family financial circumstances change (or don't change) over the course of therapy. Information from families like yours will help us achieve this goal.

This follow-up survey takes about 10 minutes to complete and includes questions about housing, utilities (heat/electricity), food and income. We understand that some of these questions may feel personal and difficult to answer. You may skip any questions and even stop completing the survey at any time.

Survey responses will be kept confidential and not shared with your treatment team. No information will be presented in any way that would permit identification of your family or child.

A. Demographics

In the following questions we would like to ask you a bit about yourself and your family.

1. What is your relationship to the patient?
    a. Mother
    b. Father
    c. Legal Guardian (please specify relationship): ____________________________
    d. Other Guardian (please specify): ______________________________________

2. How old are you?
    a. Age in years: _____
    b. Prefer not to answer

3. What is your child's current health insurance:
    a. Private insurance
    b. Medicaid Children's Health Insurance Program (CHIP) (public insurance)
    c. Private insurance plus Medicaid/CHIP
    d. Uninsured/Self-pay
    e. Other (please specify; e.g. Military): ___________

B. Housing and Transportation

The following questions apply to the home in which you and your child currently live and how you get to and from the doctor or hospital.

1. What is your zip code? ____________

2. Please tell me what type of housing you and your child live in (choose the best response):
   a. Apartment
   b. House/Townhouse/Condo
   c. Mobile home/trailer
   d. Room/rented room
   e. Shelter or transitional living situation
   f. No steady place to sleep at night (e.g. temporarily staying with others, in a hotel, on the street, in the car, in a park, etc.)
   g. Other (please specify): ____________

3. Do you think you are at risk of becoming homeless?
   a. Yes
   b. No

4. How many people (including yourself and your child) are currently living in your home?
   __________ Number of people
   __________ Of these people, how many are children (<18 years old)?
   __________ Of these people, how many are adults (>=18 years old)?
   __________ Of the adults, how many bring income into the household?

5. How many bedrooms are in your home?
   __________# bedrooms

6. How many places have you and your child lived in the past year?
   __________# of places

7. During the past 6 months, was there a time when you were not able to pay the rent or mortgage on time because of financial difficulties?
   a. Yes
   b. No

8. During the past 6 months, have you and your child had to temporarily live with other people because of financial difficulties?
   a. Yes
   b. No

9. How have you been getting to the hospital or clinic for appointments? *Please choose all that apply.*
   a. Own car
   b. Rides from friends
   c. Commercial ride (e.g. Uber/Lyft)
   d. Public (bus, subway, train)
   e. Medicaid transportation benefit
   f. Not Sure/Don't know

10. In the last 6 months, has lack of reliable transportation kept you or anyone in your family from medical appointments, meetings, work or getting things needed for daily living?
   a. Yes
   b. No

C. Utilities

*The following questions ask about heat and electricity in your home **since your child's diagnosis**.*

1. During the past 6 months, has the gas/electric/oil company sent you a letter threatening to shut off the gas/electricity/oil to the house for not paying bills?
   a. Yes
   b. No
   c. Not applicable, I don't pay for gas/electric/oil

2. During the past 6 months, has the gas/electric/oil company shut off electricity or refused to deliver oil/gas for not paying bills?
   a. Yes
   b. No
   c. Not applicable, I don't pay for gas/electric/oil

3. During the past 6 months, were there any days that your home was not heated/cooled because you couldn't pay the bills?
   a. Yes
   b. No
   c. Not applicable, I don't pay for heat or electricity

4. During the past 6 months, have you had to use a cooking stove to heat your home because you couldn't pay the bills?
   a. Yes
   b. No
   c. Not applicable, I don't pay for heat

5. During the past 6 months, did you have service disconnected on your telephone (including cell phones) because you couldn't pay the bill?
   a. Yes
   b. No
   c. Not applicable, I don't pay for telephone

D. Food

*The next set of questions are about the food eaten in your household in the last 6 months, and whether you were able to afford the food you need. Please answer these questions thinking about **the time since child was diagnosed with cancer.***

*I'm going to read you two statements that other people have made about their food situation. For each statement, please tell me whether the statement was often true, sometimes true, or never true for your household **in the last 6 months.***

1. The first statement is, "We worried whether our food would run out before we got money to buy more." Was that often, sometimes, or never true for your household in the last 6 months?
   a. Often true
   b. Sometimes true
   c. Never true
   d. Don't know or prefer not to answer

2. "The food that we bought just didn't last, and we didn't have money to get more." Was that often, sometimes, or never true for your household in the last 6 months?
   a. Often true
   b. Sometimes true
   c. Never true
   d. Don't know or prefer not to answer

E. Finances

The next set of questions are about the resources supporting your child. Please answer these questions thinking about your household ***over the last 6 months of your child's cancer treatment.***

1. Please provide your best estimate of your total combined household income (i.e. the amount your family would report on your taxes). This should include income from all sources: wages, child support payments, rent from properties, social security, disability and/or veteran's benefits, unemployment benefits, workman's compensation, and so on:
   a. $ __________
   b. Not sure or prefer not to answer

2. Please estimate about how much yearly income your family has lost due to work/employment changes (e.g. reduced hours or stopping work)?
   a. $ __________ (best estimate, can include "none")
   b. Not sure or prefer not to answer

3. Think about the past 6 months. Would you say that each month you generally ended up with:
   a. More than enough money left over
   b. Some money left over
   c. Just enough to make ends meet
   d. Almost enough to make ends meet
   e. Not enough to make ends meet

4. If you lost all of your current sources of income (for example, your paycheck, Social Security or pension, public assistance) and had to live off of your savings, how long could you continue to live at your current address and standard of living?
   a. Less than 1 month
   b. 1-2 months
   c. 3-6 months
   d. 7-12 months
   e. More than 1 year
   f. Don't know or prefer not to answer

F. Current Resources

*The next set of questions are about the resources supporting your child. Please answer these questions thinking about your household **since your child's diagnosis.***

1. Many families are able to find financial support from existing resources. Please tell us if your family is currently receiving any of the following supports?

   a. Medicaid health insurance for your child
   a. Yes
   b. No
   c. I don't know

   b. Food stamps (i.e. SNAP)
   a. Yes
   b. No
   c. I don't know

   c. Free school lunch
   a. Yes
   b. No
   c. I don't know
   d. Does not apply (my child/children is/are not in school)

   d. Food from a food pantry (sometimes called a food bank or soup kitchen)
   a. Yes
   b. No
   c. I don't know

   e. Energy assistance (discounted gas/electric bills such as LIHEAP or utility discounts)
   a. Yes
   b. No
   c. I don't know

   f. Disability benefits (i.e. SSI, Supplemental Security Income)
   a. Yes
   b. No
   c. I don't know

   g. Low-income or subsidized housing (sometimes called Section 8 housing)
   a. Yes
   b. No
   c. I don't know

G. Parent Well-Being

The following questions are about your health and well-being

1. In general, would you say your health is:
    a. Excellent
    b. Very good
    c. Good
    d. Fair
    e. Poor

2. For how long (if at all) has your health limited you in each of the following activities?

	Limited for more than 3 months	Limited for 3 months or less	Not limited at all
The kinds or amounts of **vigorous** activities you can do, like lifting heavy objects, running or participating in strenuous sports	1	2	3
The kinds or amounts of **moderate** activities you can do, like moving a table, carrying groceries, or bowling	1	2	3
Walking uphill or climbing a few flights of stairs	1	2	3
Bending, lifting, or stooping	1	2	3
Walking one block	1	2	3
Eating, dressing, bathing, or using the toilet	1	2	3

Any additional comments:

3. How much bodily pain have you had during the past 4 weeks?
    a. None
    b. Very mild
    c. Mild
    d. Moderate
    e. Severe
    f. Very severe

4. Does your health keep you from working at a job, doing work around the house, or going to school?
    a. Yes, for more than 3 months
    b. Yes, for 3 months or less
    c. No

5. Have you been unable to do certain kinds or amounts of work, housework, or schoolwork because of your health?
    a. Yes, for more than 3 months
    b. Yes, for 3 months or less
    c. No

6. For each of the following questions, please choose the answer that comes closest to the way you have been feeling during the past month:

	None of the time	A little of the time	Some of the time	A good bit of the time	Most of the time	All of the time
How much of the time has your **health limited your social activities** (like visiting with friends or close relatives)?	1	2	3	4	5	6
How much of the time have you been **a very nervous person**?	1	2	3	4	5	6
How much of the time have you felt **calm and peaceful?**	1	2	3	4	5	6
How much of the time have you felt **downhearted and blue?**	1	2	3	4	5	5
How much of the time have you been **a happy person?**	1	2	3	4	5	5
How often have you felt so **down in the dumps that nothing could cheer you up?**	1	2	3	4	5	6

Any additional comments:

____

____

7. For the following statements, please choose the option that best indicates how true or false they are for you.

	Definitely True	Mostly True	Not Sure	Mostly False	Definitely False
I am somewhat ill	1	2	3	4	5
I am as healthy as anybody I know	1	2	3	4	5
My health is excellent	1	2	3	4	5
I have been feeling bad lately	1	2	3	4	5

Any additional comments:

8. Over the last 2 weeks, how often have you been bothered by the following problems?

	Not at all	Several Days	More than half the days	Nearly Every Day
Feeling nervous, anxious or on edge	○	○	○	○
Not being able to stop or control worrying	○	○	○	○
Worrying too much about different things	○	○	○	○
Trouble relaxing	○	○	○	○
Being so restless that it is hard to sit still	○	○	○	○
Becoming easily annoyed or irritable	○	○	○	○
Feeling afraid as if something awful might happen	○	○	○	○

Any additional comments:

H. Parent-Patient Healthcare Activation

The following questions ask about your levels of knowledge, skills, and confidence in managing your child's health. For each question, please choose the option that best indicates how much you agree with the statement as it applies to you on average.

	Strongly Disagree	Disagree	Agree	Strongly Agree
1. When all is said and done, I am the person who is responsible for taking care of my child's health	1	2	3	4
2. Taking an active role in my child's health care is the most important thing that affects his/her health	1	2	3	4
3. I am confident I can help prevent or reduce problems associated with my child's health	1	2	3	4
4. I know what each of my child's immunizations are for	1	2	3	4
5. I am confident that I can tell when I need to go get medical care and when I can handle my child's health problem myself	1	2	3	4
6. I am confident I can tell a doctor concerns I have about my child's health, even when he or she does not ask	1	2	3	4
7. I am confident that I can follow through on medical treatments I need to do for my child at home	1	2	3	4
8. I understand my child's health problems and what causes them	1	2	3	4
9. I know what treatments are available for my child's health	1	2	3	4
10. I have been able to help my child maintain (keep up with) recommended changes like eating right or exercising	1	2	3	4
11. I know how to prevent problems with my child's health	1	2	3	4

12. I am confident I can figure out solutions when new situations arise with my child's health	1	2	3	4
13. I am confident I can help my child maintain changes, like eating right and exercise, even during times of stress	1	2	3	4

Any additional comments:

I. Assessment

We would like to improve the support we provide families during therapy and welcome your feedback on what has helped or not helped your family over the past six months. The below questions are intended to help us understand how well-supported your family has been with regards to day to day basic needs at home (such as food, heat/electricity, rent/mortgage, transportation).

1. How satisfied were you with the support you received from [PediCARE/your team]?
    a. Very satisfied
    b. Somewhat satisfied
    c. Somewhat dissatisfied
    d. Very dissatisfied

2. Over the past 6 months, did you want any assistance from the hospital or your care team with any of the following basic household needs?
    a. Food
        a. Yes
        b. No
        c. Don't know/prefer not to answer
    b. Rent or mortgage
        a. Yes
        b. No
        c. Don't know/prefer not to answer
    c. Utilities (e.g. heat or electricity)
        a. Yes
        b. No
        c. Don't know/prefer not to answer
    d. Transportation to and from the hospital
        a. Yes
        b. No
        c. Don't know/prefer not to answer
    e. Other: (please specify) _____________

3. If yes to any of the above, did you receive any assistance with the following?
    a. Food
        a. Yes
        b. No
        c. Don't know/prefer not to answer

- b. Rent or mortgage
    - a. Yes
    - b. No
    - c. Don't know/prefer not to answer
- c. Utilities (e.g. heat or electricity)
    - a. Yes
    - b. No
    - c. Don't know/prefer not to answer
- d. Transportation to and from the hospital
    - a. Yes
    - b. No
    - c. Don't know/prefer not to answer
- e. Other: (please specify) ________

4. If yes, would you say the assistance you received was:
    - a. Just right
    - b. Helpful but not enough
    - c. Not nearly enough to be helpful
    - d. Don't know/prefer not to answer

[PediCARE Recipients only]

5. How likely would you be to recommend participation in the PediCARE support program for other families undergoing childhood cancer treatment?
    a. Very likely
    b. Somewhat likely
    c. Somewhat unlikely
    d. Very unlikely
    e. Don't know/prefer not to answer

6. Did the PediCARE support program make it easier to meet any of the below basic needs (choose all that apply)?
    a. No
    b. Yes, easier to buy food for my family
    c. Yes, easier to get to and from hospital/clinic

7. How much of the food money provided would you say you used each month?
    a. All
    b. Most
    c. Some
    d. Very little
    e. None

8. If you did not use most/all of the food money, what were the main reasons?
    a. Not applicable
    b. Didn't need it
    c. Forgot about it
    d. Hard to use
    e. Saving money for if finances get harder
    f. I don't know
    g. Other (please specify): ___________________

9. If you used any of the food money provided, how easy or difficult was it to buy groceries for your family with this approach?
    a. Not applicable
    b. Very easy
    c. Somewhat easy
    d. Somewhat difficult
    e. Very difficult

10. About how many of the 8 roundtrip Ride Health rides provided each month would you say you used?
    a. Not applicable
    b. None
    c. 1 - 2
    d. 3 - 4
    e. 4 - 5
    f. 5 - 6
    g. 6 - 7
    h. 7 - 8

11. If you did not use all of the Ride Health rides, what were the main reasons?
    a. Not applicable
    b. Didn't need it
    c. Forgot about it
    d. Hard to use
    e. Didn't like this type of transportation
    f. I don't know
    g. Other (please specify): _____________

12. How much of the gas card money provided would you say you used each month?
    a. Not applicable
    b. All
    c. Most
    d. Some
    e. Very little
    f. None

13. If you did not use most/all of the gas cards, what were the main reasons?
    a. Not applicable
    b. Didn't need it
    c. Forgot about it
    d. Hard to use
    e. Saving money for if finances get harder
    f. I don't know
    g. Other (please specify): _________________

14. Would you say the PediCARE program helped you address other basic needs at home (such as heat or electricity bills, rent or mortgage)?
    a. Definitely
    b. Somewhat
    c. Not at all

15. If the PediCARE program were to be redesigned and what types of other supports would be more helpful? ____

We have come to the end of the questionnaire.

To be completed by administering research staff.

Today's date (month/day/year): __ __ / __ __ / __ __ __ __

Site at which survey administered:

a. DFCI
b. UAB

Name of staff member administering survey (Last, First): ______________________

Survey administered:

a. English, US
b. Spanish, US

Interpreter used:

a. Yes (specify language): _____________
b. No

Appendix C. Instacart Information Sheet

- Instacart customer service: Call **(888)-246-7822 (English and Spanish)**

Redeeming Your Instacart Gift Card

1. Open your email inbox & open the email from Instacart "PediCARE has sent you a gift card"
2. Click the link in the email that says "redeem gift card." This will open the Instacart website and the gift card money will automatically be put into your Instacart account.
3. This money belongs to you and your account both during and after the PediCARE study.

Ordering Groceries on Instacart

1. **Login to your Instacart account:** www.instacart.com
   a. Use this email address: ____________________
   b. Use this password: ______________________
2. **Pick your grocery store**
   a. You can buy groceries from any store listed at the top of your account
   b. You can buy groceries from multiple stores in one order (each store has an "order minimum")

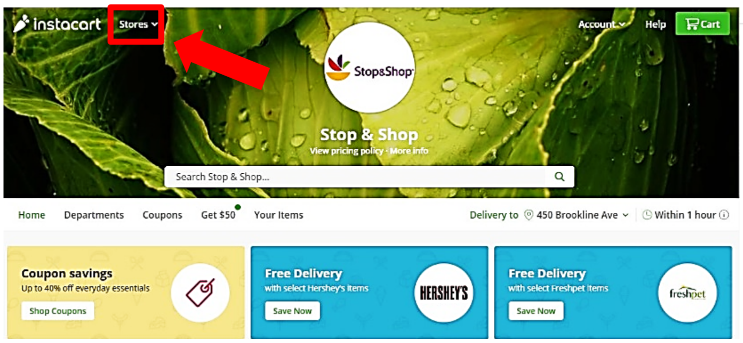

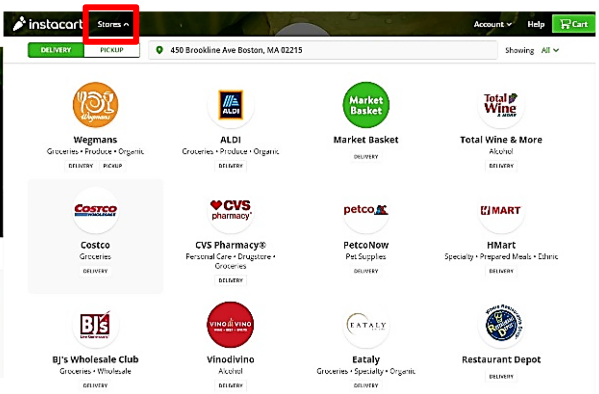

3. **Choose your groceries**
   a. Click on "Departments" to browse different groceries (just like aisles in the store).
   b. Search for specific items (like: "Kraft Mac n Cheese") in the search bar.
   c. Add items to your cart—click on the green (+) sign on the top-right corner of item
   d. To checkout, click "Cart" in the top right corner of the page and then click "Checkout."
4. **Choose where & when you want groceries delivered**
   a. Choose the address
   b. Choose the day and time under "Delivery Time"
   c. Enter a phone number that your delivery person can call/text with updates/questions
5. **Buy your groceries**
   a. Your gift card money will be automatically applied to your checkout basket
      - It will say "Credit/Discount applied"
      - You do NOT need to add a promo code
   b. Choose how much money to tip your driver
      - Click the green "Change" button next to "Driver Tip" in your order summary
   c. Click the green "Place Order" button to complete your order

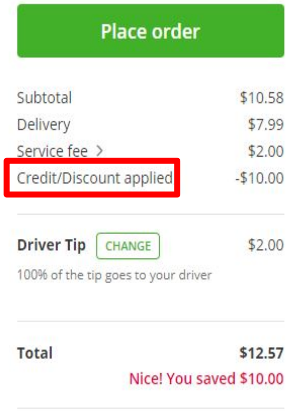

6. **At any time: Check how much Instacart gift card money you have left**
   a. Login to your Instacart account: www.instacart.com
   b. Click **"Account"** → **"Your account"** → **"Promo codes"** in the left-hand menu
   c. The remaining amount of money will be shown as "Available Credit/Discount"

Appendix D: Data Safety Monitoring Plan

DFCI IRB Protocol #: 18-294

APPENDIX D

Dana-Farber/Harvard Cancer Center
Multi-Center Data and Safety Monitoring Plan

TABLE OF CONTENTS

1. INTRODUCTION 68
1.1 Purpose 68
1.2 Multi-Center Data and Safety Monitoring Plan Definitions 68
2. GENERAL ROLES AND RESPONSIBILITIES 69
2.1 DF/HCC Sponsor 69
2.2 Coordinating Center 70
2.3 Participating Institution 70
3. DF/HCC REQUIREMENTS FOR MULTI-CENTER PROTOCOLS 71
3.1 Protocol Distribution 71
3.2 Protocol Revisions and Closures 71
3.3 Informed Consent Requirements 72
3.4 IRB Documentation 72
3.5 IRB Re-Approval 72
3.6 Participant Confidentiality and Authorization Statement 72
3.7 DF/HCC Multi-Center Protocol Registration Policy 73
3.8 DF/HCC Protocol Case Number/Study ID 73
3.9 Safety Assessments and Toxicity Monitoring 74
4. MONITORING: QUALITY CONTROL 75
4.1 Ongoing Monitoring of Protocol Compliance 75
4.2 Monitoring Reports 76
4.3 Accrual Monitoring 76
5. AUDITING: QUALITY ASSURANCE 76
5.1 DF/HCC Internal Audits 77
5.2 Audit Notifications 77
5.3 Audit Reports 77
5.4 Participating Institution Performance 77

INTRODUCTION

The Dana-Farber/Harvard Cancer Center Multi-Center Data and Safety Monitoring Plan (DF/HCC DSMP) outlines the procedures for conducting a DF/HCC Multi-Center research protocol. The DF/HCC DSMP serves as a reference for any sites external to DF/HCC that are participating in a DF/HCC clinical trial.

Purpose

To establish standards that will ensure that a Dana-Farber/Harvard Cancer Center Multi-Center protocol will comply with Federal Regulations, Health Insurance Portability and Accountability Act (HIPAA) requirements and applicable DF/HCC Standard Operating Procedures.

Multi-Center Data and Safety Monitoring Plan Definitions

DF/HCC Multi-Center Protocol*:* A research protocol in which one or more outside institutions are collaborating with Dana-Farber/Harvard Cancer Center where a DF/HCC investigator is the sponsor. DF/HCC includes Dana-Farber/Partners Cancer Care (DF/PCC) Network Clinical Trial Affiliates.

Lead Institution: One of the Dana-Farber/Harvard Cancer Center consortium members (Dana-Farber Cancer Institute (DFCI), Massachusetts General Hospital (MGH), Beth Israel Deaconess Medical Center (BIDMC), Boston Children's Hospital (BCH), Brigham and Women's Hospital (BWH) responsible for the coordination, development, submission, and approval of a protocol as well as its subsequent amendments per the DFCI IRB and applicable regulatory guidelines (CTEP, Food and Drug Administration (FDA), Office of Biotechnology Activities (OBA) etc.). The Lead Institution is typically the home of the DF/HCC Sponsor. The Lead Institution also typically serves as the Coordinating Center for the DF/HCC Multi-Center Protocol.

DF/HCC Sponsor: The person sponsoring the submitted Multi-Center protocol who takes responsibility for initiation, management and conduct of the protocol at all research locations. In applicable protocols, the DF/HCC Sponsor will serve as the single liaison with any regulatory agencies (i.e. CTEP Protocol and Information Office (PIO), FDA, OBA etc.). The DF/HCC Sponsor has ultimate authority over the protocol and is responsible for the conduct of the study at DF/HCC and all Participating Institutions. In most cases the DF/HCC Sponsor is the same person as the DF/HCC Overall Principal Investigator; however, both roles can be filled by two different people. For CTEP Protocols this person is called the Protocol Chair.

Participating Institution: An institution that is outside the DF/HCC and DF/PCC consortium that is collaborating with DF/HCC on a protocol where the sponsor is a DF/HCC Investigator. The Participating Institution acknowledges the DF/HCC Sponsor as having the ultimate authority and responsibility for the overall conduct of the study.

Coordinating Center: The entity (i.e. Lead Institution, Medical Monitor, Contract Research Organization (CRO), etc) that provides administrative support to the DF/HCC Sponsor in order

that he/she may fulfill the responsibilities outlined in the protocol document and DSMP, and as specified in applicable regulatory guidelines (i.e. CTEP Multi-Center Guidelines). In general, the Lead Institution is the Coordinating Center for the DF/HCC Multi-Center Protocol.

DF/HCC Office of Data Quality (ODQ): A group within DF/HCC responsible ensuring high-quality standards are used for data collection and the ongoing management of clinical trials, auditing, and data and safety monitoring. ODQ also coordinates quality assurance efforts related to multi-center clinical research.

DF/HCC Clinical Trials Research Informatics Office (CTRIO): A group within DF/HCC responsible for providing a comprehensive data management platform for managing clinical trial data.

GENERAL ROLES AND RESPONSIBILITIES

For DF/HCC Multi-Center Protocols, the DF/HCC Sponsor, the Coordinating Center, and the Participating Institutions are expected to adhere to the following general responsibilities:

DF/HCC Sponsor

The DF/HCC Sponsor, Dr. Kira Bona, will accept responsibility for all aspects of conducting a DF/HCC Multi-Center protocol which includes but is not limited to:

- Oversee the coordination, development, submission, and approval of the protocol as well as subsequent amendments.
- Ensure that the investigators, study team members, and Participating Institutions are qualified and appropriately resourced to conduct the protocol.
- Include the Multi-Center Data and Safety Monitoring Plan as an appendix to the protocol.
- Ensure all Participating Institutions are using the correct version of the protocol.
- Ensure that each participating investigator and study team member receives adequate protocol training (and/or a Site Initiation Visit prior to enrolling participants) and throughout trial’s conduct as needed.
- Ensure the protocol will be provided to each participating site in a language understandable to all applicable site personnel when English is not the primary language.
- Monitor progress and overall conduct of the study at all Participating Institutions.
- Ensure all DFCI Institutional Review Board (IRB), DF/HCC and other applicable reporting requirements are met.
- Review data and maintain timely submission of data for study analysis.
- Ensure compliance with all requirements as set forth in the Code of Federal Regulations, applicable DF/HCC requirements, HIPAA requirements, and the approved protocol.

- Commit to the provision that the protocol will not be rewritten or modified by anyone other than the DF/HCC Sponsor.
- Identify and qualify Participating Institutions and obtain accrual commitments prior to extending the protocol to that site.
- Monitor accrual and address Participating Institutions that are not meeting their accrual requirements.

Coordinating Center

The general responsibilities of the Coordinating Center may include but are not limited to:

- Assist in protocol development.
- Review registration materials for eligibility and register participants from Participating Institutions in the DF/HCC clinical trial management system (CTMS).
- Distribute protocol and informed consent document updates to Participating Institutions as needed.
- Oversee the data collection process from Participating Institutions.
- Maintain documentation of deviations/violation submitted by Participating Institutions and provide to the DF/HCC Sponsor for timely review and submission to the DFCI IRB, as necessary.
- Provide Participating Institutions with information regarding DF/HCC requirements that they will be expected to comply with.
- Carry out plan to monitor Participating Institutions either by on-site or remote monitoring.
- Maintain Regulatory documents of all Participating Institutions which includes but is not limited to the following: local IRB approvals/notifications from all Participating Institutions, confirmation of Federalwide Assurances (FWAs) for all sites, Screening Logs for all sites, IRB approved consents for all sites
- Conduct regular communications with all Participating Institutions (conference calls, emails, etc) and maintain documentation all relevant communications.

Participating Institution

Each Participating Institution is expected to comply with all applicable federal regulations and DF/HCC requirements, the protocol and HIPAA requirements.

The general responsibilities for each Participating Institution may include but are not limited to:

- Document the delegation of research specific activities to study personnel.
- Commit to the accrual of participants to the protocol.
- Submit protocol and/or amendments to their local IRB of record.
- Maintain regulatory files as per sponsor requirements.
- Provide the Coordinating Center with regulatory documents or source documents as requested.

- Participate in protocol training prior to enrolling participants and throughout the trial as required (i.e. teleconferences).
- Update Coordinating Center with research staff changes on a timely basis.
- Submit protocol deviations and violations to local IRB per institutional requirements and to the DF/HCC Sponsor in accordance with DF/HCC requirements.
- Have office space, office equipment, and internet access that meet HIPAA standards.
- Participate in any quality assurance activities and meet with monitors or auditors at the conclusion of a visit to review findings.
- Promptly provide follow-up and/or corrective action plans for any monitoring queries or audit findings.

DF/HCC REQUIREMENTS FOR MULTI-CENTER PROTOCOLS

The following section will clarify DF/HCC Requirements and further detail the expectations for participating in a DF/HCC Multi-Center protocol.

Protocol Distribution

The Coordinating Center will distribute the final DFCI IRB approved protocol and any subsequent amended protocols to all Participating Institutions.

Protocol Revisions and Closures

The Participating Institutions will receive notification of protocol revisions and closures from the Coordinating Center. It is the individual Participating Institution's responsibility to notify its IRB of these revisions.

- **Non life-threatening revisions:** Participating Institutions will receive written notification of protocol revisions regarding non life-threatening events from the Coordinating Center. Non-life-threatening protocol revisions must be IRB approved and implemented within 90 days from receipt of the notification.

- **Revisions for life-threatening causes:** Participating Institutions will receive immediate notification from the Coordinating Center concerning protocol revisions required to protect lives with follow-up by fax, mail, e-mail, etc. Life-threatening protocol revisions will be implemented immediately followed by IRB request for approval.

- **Protocol closures and temporary holds:** Participating Institutions will receive notification of protocol closures and temporary holds from the Coordinating Center. Closures and holds will be effective immediately. In addition, the Coordinating Center, will update the Participating Institutions on an ongoing basis about protocol accrual data so that they will be aware of imminent protocol closures.

Informed Consent Requirements

The DF/HCC approved informed consent document will serve as a template for the informed consent for Participating Institutions. The Participating Institution consent form must follow the consent template as closely as possible and should adhere to specifications outlined in the DF/HCC Guidance Document on Model Consent Language for Investigator-Sponsored Multi-Center Trials. This document will be provided separately to each Participating Institution upon request.

Participating Institutions are to send their version of the informed consent document and HIPAA authorization, if a separate document, to the Coordinating Center for review and approval prior to submission to their local IRB. The approved consent form must also be submitted to the Coordinating Center after approval by the local IRB for all consent versions.

IRB Documentation

The DFCI IRB will act as the single IRB (sIRB) of record for this study (see protocol section 6).

The following must be on file with the Coordinating Center:

- Initial approval letter of the Participating Institution's IRB.
- Copy of the Informed Consent Form(s) approved by the Participating Institution’s IRB.
- Participating Institution’s IRB approval for all amendments.
- Annual approval letters by the Participating Institution's IRB.

IRB Re-Approval

Verification of IRB re-approval from the Participating Institutions is required in order to continue research activities. There is no grace period for continuing approvals.

The Coordinating Center will not register participants if a re-approval letter is not received from the Participating Institution on or before the anniversary of the previous approval date.

Participant Confidentiality and Authorization Statement

In 1996, congress passed the first federal law covering the privacy of health information known as the Health Insurance Portability and Accountability Act (HIPPA). Any information, related to the physical or mental health of an individual is called Protected Health Information (PHI). HIPAA outlines how and under what circumstances PHI can be used or disclosed.

In order for covered entities to use or disclose protected health information during the course of a study, the study participant must sign an authorization statement. This authorization statement may or may not be separate from the informed consent document. The Coordinating

Center, with the approval from the DFCI IRB, will provide a consent template, with information regarding authorization for the disclosure of protected health information.

The DF/HCC Sponsor will use all efforts to limit its use of protected health information in its trials. However, because of the nature of these trials, certain protected health information must be collected. DF/HCC has chosen to use authorizations, signed by the participant in the trial, rather than limited data sets with data use agreements.

DF/HCC Multi-Center Protocol Confidentiality

All documents, investigative reports, or information relating to the participant are strictly confidential. Whenever reasonably feasible, any participant specific reports submitted to the Coordinating Center should be de-identified. It is recommended that the assigned protocol case number (as described below) be used for all participant specific documents. Participant initials may be included or retained for cross verification of identification.

DF/HCC Multi-Center Protocol Registration Policy

Participant Registration and Randomization

Please see Section 5.3 of the protocol for instructions on the process to register and randomize patients.

Eligibility Exceptions

No exceptions to the eligibility requirements for a protocol without DFCI IRB approval will be permitted. All Participating Institutions are required to fully comply with this requirement. The process for requesting an eligibility exception is defined below.

DF/HCC Protocol Case Number/Study ID

At the time of registration, the following identifiers are required for all subjects: initials, date of birth, gender, race and ethnicity. Once eligibility has been established and the participant successfully registered, the participant is assigned a unique protocol case number/study ID. Participating Institutions should submit all de-identified subsequent communication and documents to the Coordinating Center, using this case number to identify the subject.

Protocol Deviations, Exceptions and Violations

Federal Regulations require an IRB to review proposed changes in a research activity to ensure that researchers do not initiate changes in approved research without IRB review and approval, except when necessary to eliminate apparent immediate hazards to the participant. DF/HCC requires all departures from the defined procedures set forth in the IRB approved protocol to be reported to the DF/HCC Sponsor, who in turn is responsible for reporting to the DFCI IRB.

For reporting purposes, DF/HCC uses the terms "violation", "deviation" and "exception" to describe departures from a protocol. All Participating Institutions must adhere to these requirements for reporting to the DF/HCC Sponsor and will follow their institutional policy for reporting to their local IRB.

Definitions

Protocol Deviation: Any departure from the defined procedures set forth in the IRB-approved protocol which is *prospectively approved* prior to its implementation.

Protocol Exception: Any protocol deviation that relates to the eligibility criteria, e.g. enrollment of a participant who does not meet all inclusion/exclusion criteria.

Protocol Violation: Any protocol departure that was not *prospectively approved* by the IRB prior to its initiation or implementation.

Reporting Procedures

DF/HCC Sponsor: is responsible for ensuring that clear documentation is available in the medical record and/or regulatory documents to describe all protocol exceptions, deviations and violations. The DF/HCC Sponsor will also be responsible for ensuring that all protocol violations/deviations are promptly reported per DFCI IRB guidelines.

Participating Institutions: Protocol deviations require prospective approval from the DFCI IRB. The Participating Institution must submit the deviation request to the Coordinating Center who will then submit the deviation request to the DFCI IRB. Upon DFCI IRB approval the deviation is submitted to the Participating Institution IRB, per institutional policy. A copy of the Participating Institution's IRB report and determination will be forwarded to the Coordinating Center within 10 business days after the original submission. The deviation may not be implemented without all required approvals.

All protocol violations must be sent to the Coordinating Center in a timely manner. The Coordinating Center will provide training for the requirements for the reporting of violations.

Coordinating Center: Upon receipt of the violation/deviation report from the Participating Institution, the Coordinating Center will submit the report to the DF/HCC Sponsor for review. Subsequently, the Participating Institution's IRB violation/deviation report will be submitted to the DFCI IRB for review per DFCI IRB reporting guidelines.

Safety Assessments

The study teams at all participating institutions are responsible for protecting the safety, rights and well-being of study participants.

MONITORING: QUALITY CONTROL

The quality control process for a clinical trial requires verification of protocol compliance and data accuracy. The Coordinating Center, with the aid of the DF/HCC Office of Data Quality, provides quality control oversight for the protocol.

Ongoing Monitoring of Protocol Compliance

This is a small pilot multisite clinical trial of a supportive care intervention that provides minimal risk to participants and the prospect of direct benefit. As such, data monitoring will be carried out by a Data Safety Monitoring Committee (DSMC) composed of investigators representing different disciplines and expertise. The team will be comprised of Co-investigators: Drs. Bona and Wolfson; as well as three members who are not participating in the study (Dr. Joanne Wolfe; Dr. Anna Muriel; Dr. Abby Rosenberg) who will act as independent monitors. The committee will be convened at the beginning of the study via conference calls, to provide input and guidance on study conduct including quality assurance and safety issues as well as data management activities. All data reviewed by the DSMC will be blinded. DSMC virtual meetings will take place quarterly by videoconference calls.

The DSMC will be in charge of monitoring: 1) data quality for completeness, timeliness, and accuracy including conformance with informed consent requirements; 2) participants' accrual and retention; 3) adverse events; 4) participants' withdrawals, compliance issues or any complaints about the research; 5) intervention fidelity indicators; 6) compliance with data management procedures; 7) reviewing any protocol modifications/deviations that may occur between DSMC review periods.

Safety Monitoring: As above, we will utilize a DSMC to ensure that the trial is conducted according to the approved protocol. There are no physical risks to this study. There is a minimal risk of psychological distress to parents from completing in-depth interviews and surveys on the sensitive topic of basic resource needs and finances.

Data Management: Registration and patient tracking will be performed using DF/HCC ODQ OnCore system. This protocol will be registered with OnCore, allowing for centralized enrollment tracking in an ODQ-approved manner. The study team has been trained and has experience in using this system. All surveys and abstraction data will be stored in an institutionally-coordinated Research Electronic Data Capture (REDCap) database, which offers a secure web application. An institution-specific tracking log will also be kept as an Excel spreadsheet. These spreadsheets will be located on HIPAA-compliant DF/HCC servers. Physical study documents (i.e. paper surveys) will be stored in locked cabinets, and will only be identified by study ID.

Study data from all sites will be entered, stored, and managed in a password-protected REDCap database structured for this study on a secured server. REDCap is a research-focused electronic data capture system. Data will be verified both at point-of-entry and subsequently by a series of range checks, logical between-items checks, and outlier checks.

Quality Control Monitoring: The quality control process for a clinical trial requires verification of protocol compliance and data accuracy. The Coordinating Center (DFCI), with the aid of the ODQ provides quality control oversight for the protocol. Accrual will be monitored for each participating institution by the DSMC.

The Participating Institutions may be required to submit participant source documents to the Coordinating Center for monitoring. Participating Institution may also be subject to on-site monitoring conducted by the Coordinating Center.

Participating institutions will be required to participate in monthly Coordinating Center initiated teleconferences.

Remote Monitoring will be performed by central monitoring of REDCap survey completion (data are electronically entered and stored thus source verification is non-applicable unless paper/pencil survey administered due to technological challenges in which case these will be scanned and securely emailed to DFCI for central data entry and source documentation).

Monitoring Reports

The DF/HCC Sponsor will review all monitoring reports to ensure protocol compliance. The DF/HCC Sponsor may increase the monitoring activities at Participating Institutions that are unable to comply with the protocol, DF/HCC Sponsor requirements or federal and local regulations.

Accrual Monitoring

Prior to extending a protocol to an external site, the DF/HCC Sponsor will establish accrual requirements for each participating institution. Accrual will be monitored for each participating institution by the DF/HCC Sponsor or designee. Sites that are not meeting their accrual expectations may be subject to termination.

Accrual expectations for University Alabama Birmingham are as follows: Enrollment of 12 participants over 12 months.

AUDITING: QUALITY ASSURANCE

Auditing is a method of Quality Assurance and involves the systematic and independent examination of all trial related activities and documents. Audits determine if evaluated activities were appropriately conducted and whether data was generated, recorded and analyzed, and accurately reported per the protocol, applicable Policies, and the Code of Federal Regulations (CFR).

DF/HCC Internal Audits

All Participating Institutions are subject to audit by the DF/HCC Office of Data Quality (ODQ). Typically, approximately 3-4 participants would be audited at the site over a 2-day period. If violations which impact participant safety or the integrity of the study are found, more participant records may be audited.

Audit Notifications

It is the Participating Institution's responsibility to notify the Coordinating Center of all external audits or inspections (e.g., FDA, EMA, NCI) that involve this protocol. All institutions will forward a copy of final audit and/or re-audit reports and corrective action plans (if applicable) to the Coordinating Center, within 12 weeks after the audit date.

Audit Reports

The DF/HCC Sponsor will review all final audit reports and corrective action plans, if applicable. The Coordinating Center, must forward any reports to the DF/HCC ODQ per DF/HCC policy for review by the DF/HCC Audit Committee. For unacceptable audits, the DF/HCC Audit Committee would forward the final audit report and corrective action plan to the DFCI IRB as applicable.

Participating Institution Performance

The DF/HCC Sponsor and the IRB of record are charged with considering the totality of an institution's performance in considering institutional participation in the protocol.

Participating Institutions that fail to meet the performance goals of accrual, submission of timely and accurate data, adherence to protocol requirements, and compliance with state and federal regulations, may be recommended for a six-month probation period. Such institutions must respond with a corrective action plan and must demonstrate during the probation period that deficiencies have been corrected, as evidenced by the improved performance measures. Participating Institutions that fail to demonstrate significant improvement will be considered by the DF/HCC Sponsor for revocation of participation. A DF/HCC Sponsor and/or the DFCI IRB may terminate a site's participation if it is determined that a site is not fulfilling its responsibilities as described above.

Appendix E. Ride Health Information Sheet

- **Ride Health Customer Support:** (833) 267-7433 (English and Spanish).
- **PediCARE Study questions:** Contact your local study team

How to log in to Ride Health

1. Go to the Ride Health Patient portal at https://dana-farber.ride-health.com/login.
    a. Click "I am a Patient"
    b. Enter your cell phone number—you do *not* need to make an account and password.
    c. Click "Send SMS" and you will get a text with a 6-digit passcode
    d. Enter the 6-digit passcode into the portal login and click "Verify"

Ride Options

You can create a ride for (1) an appointment in the future **or** (2) right now.

a. **SCHEDULED ride:** If you know the date and time of a future appointment, you can schedule a round-trip ride between your home and the hospital/clinic
b. **ON-DEMAND ride:** if you need a ride right now or will need one soon but don't know exactly when
c. **Change/Edit ride:** (1) Cancel it & create a new one, or (2) Call the Customer Support Line

Creating a SCHEDULED Ride for the FUTURE (e.g. a planned clinic visit)

1. Click the "New Ride" button. Rides can be made up to a week ahead of time.
    - If you need a ride **to the hospital** for your appointment, choose "Appointment."
    - If you are leaving the hospital **to go home**, choose "Discharge"
    - If you need a ride **for any other reason**, pick "Other."
2. Click: "At a Scheduled Time"
3. Choose the date, addresses, and time of your appointment. Then, click "Create."
4. On the day of your scheduled ride: Ride Health will send you a reminder text
    - You can *cancel* the ride if you don't need it anymore by replying "2" to the text
5. Before your pick-up: Ride Health will text you a message listing your ride details:
    a. Driver's name
    b. Make, model, and color of the car picking you up
    c. License plate number
    d. Estimated arrival time at your location
    e. Driver's phone number

Creating an ON-DEMAND Ride for RIGHT NOW

1. Click "On-demand"
    - Choose the address where you will be picked up
    - You will receive a text to your phone that says: *"Press '1' when you are ready for your ride. Press '2' if you no longer need a ride."*
    - When you are ready to be picked up: reply "1" and a car will immediately be called for you
    - Your ride may take anywhere from 1-15 minutes to arrive

2. Before your pick-up: Ride Health will text you a message listing your ride details:
    a. Driver's name
    b. Make, model, and color of the car picking you up
    c. License plate number
    d. Estimated arrival time at your location
    e. Driver's phone number
