# Supplementary material for: Providing Groceries and Transportation to Poverty-Exposed Pediatric Oncology Families: The PediCARE Pilot Randomized Clinical Trial
Source: JAMA Netw Open. 2024 May 31;7(5):e2412890. doi: 10.1001/jamanetworkopen.2024.12890 (PMC11143457; doi:10.1001/jamanetworkopen.2024.12890)
Supplement: Supplement 2. — eAppendix. Supplemental Methods [file jamanetwopen-e2412890-s002.pdf]

Supplemental Online Content

Newman H, Jones E, Li Y, et al. Providing groceries and transportation to poverty-exposed pediatric oncology families: the PediCARE pilot randomized clinical trial. *JAMA Netw Open*. 2024;7(5):e2412890. doi:10.1001/jamanetworkopen.2024.12890

eAppendix. Supplemental Methods

This supplemental material has been provided by the authors to give readers additional information about their work.

© 2024 Newman H et al. *JAMA Network Open.*

eAppendix. Supplemental Methods

Detailed Study Methods

Inclusion and Exclusion Criteria

Children diagnosed with cancer at DFCI and UAB served as the study cohort for PediCARE with parents/guardians as survey informants and intervention recipients.

Inclusion criteria:
1) Child initiated chemotherapy in the last 2 months for newly diagnosed cancer
2) Planned receipt of at least 4 cycles of chemotherapy at DFCI or UAB
3) Parent/guardian screened positive for HMH*
4) Child is <18 years at time of enrollment

* In accordance with previous research families were operationalized as having HMH for eligibility purposes if they reported at least one of the below four concrete needs: (1) Food insecurity. (2) Housing Insecurity. (3) Energy Insecurity. (4) Transportation Insecurity. Families are considered to have insecurity in a domain if they screen positive to at least one survey item in that domain. HMH screening is performed as standard of care by site-specific providers or will be conducted by a member of the research study team.

Exclusion criteria:
1) Child with diagnosis of relapsed cancer
2) Child planned to receive fewer than 4 cycles of chemotherapy
3) Child planned to receive observation, radiation or surgical resection only
4) Planned transfer of child to a non-DFCI or UAB facility for chemotherapy treatment
5) Foreign national family receiving cancer care as an Embassy-pay patient
6) Child is enrolled on DFCI 16-001 (due to ongoing embedded descriptive HMH study) or NA if enrolling at UAB
7) Child is enrolled on ANBL1531 or AALL1731 (due to ongoing embedded descriptive HMH studies)

Study design and participants

Eligible families were approached in-person during a scheduled clinic visit or inpatient hospital stay to offer consent to participation. Baseline and post-intervention surveys were available paper-pencil or on a tablet in English or Spanish and were administered in any language with the assistance of an interpreter by a trained clinical research coordinator (CRC). Survey domains included: sociodemographics, HMH, finances, and utilization of means-tested governmental resources. Surveys were preferentially administered face-to-face with questions read aloud by a CRC to minimize health literacy barriers and took approximately 10-15 minutes to complete. Surveys were conducted by telephone, virtual platform, or self-completed hard copy if in-person administration was not feasible. The 6-month follow up survey was completed by the same guardian as baseline survey with a CRC in clinic or the inpatient setting. Participants received a gift card following each survey. A trained CRC abstracted patient age, cancer type, and clinical trial enrollment status from the medical record. Ten percent of records were reviewed for quality control.

© 2024 Newman H et al. *JAMA Network Open.*

Treatment Arms

Intervention (*usual supportive care plus PediCARE)*

Groceries were provided via Instacart, an online grocery-delivery platform which is available in a majority of US zip codes. Participants received monthly gift cards electronically delivered into an Instacart account in a standardized dollar amount according to family size based on the US Department of Agriculture (USDA) Thrifty Food Plan which serves as the basis for food stamps. Grocery purchasing was at the discretion of participants, with no restrictions. Transportation was provided via participant choice of (1) HIPAA-compliant, patient-scheduled ride-share via the non-emergency medical transportation company Ride Health, or (2) gas cards in a standardized dollar amount based on distance from home to hospital for individuals with access to a vehicle.

Control (*usual supportive care)*

Participants in both study arms received institution-specific usual supportive care, consistent with the standard of care in pediatric oncology. Usual care pertinent to social determinants of health varies nationally, but frequently includes: 1) psychosocial clinician support; 2) social work screening for social and financial needs; 3) social work assistance with applications for financial supports on a case-by-case basis; and 4) provision of parking vouchers or gift cards when available. All study participants received usual supportive care. Families randomized to PediCARE received the intervention as an added layer of support in addition to usual care.

Statistical analysis

Baseline characteristics including patient demographics and household, disease, and treatment characteristics were summarized using descriptive statistics. All analyses were performed using Stata (Stata, version 17.0; StataCorp).

© 2024 Newman H et al. *JAMA Network Open.*
