# Supplementary material for: Providing Groceries and Transportation to Poverty-Exposed Pediatric Oncology Families: The PediCARE Pilot Randomized Clinical Trial
Source: JAMA Netw Open. 2024 May 31;7(5):e2412890. doi: 10.1001/jamanetworkopen.2024.12890 (PMC11143457; doi:10.1001/jamanetworkopen.2024.12890)
Supplement: Supplement 3. — Data Sharing Statement [file jamanetwopen-e2412890-s003.pdf]

Data Sharing Statement

Newman. Providing Groceries and Transportation to Poverty-Exposed Pediatric Oncology Families. *JAMA Netw Open*. Published May 24, 2024.
doi:10.1001/jamanetworkopen.2024.12890

Data
Data available: Yes
Data types: Deidentified participant data
How to access data: email kira.bona@childrens.harvard.edu
When available: With publication

Supporting Documents
Document types: None

Additional Information
Who can access the data: researchers whose proposed use of the data has been approved
Types of analyses: specified research purpose
Mechanisms of data availability: after approval of a proposal
